# Leveraging GWAS data to identify metabolic pathways and networks involved in maize lipid biosynthesis

**DOI:** 10.1111/tpj.14282

**Published:** 2019-03-20

**Authors:** Hui Li, Adam Thrash, Juliet D. Tang, Linlin He, Jianbing Yan, Marilyn L. Warburton

**Affiliations:** ^1^ School of Biological Science and Technology University of Jinan Jinan 250022 China; ^2^ Institute for Genomics, Biocomputing & Biotechnology Mississippi State University MS 39762 USA; ^3^ USDA FS Forest Products Laboratory Durability and Wood Protection Starkville MS 39759 USA; ^4^ National Key Laboratory of Crop Genetic Improvement Huazhong Agricultural University Wuhan 430070 China; ^5^ USDA ARS Corn Host Plant Resistance Research Unit (CHPRRU) Mississippi State MS 39762 USA

**Keywords:** maize, lipid metabolism, pathway analysis, genome‐wide association study

## Abstract

Maize (*Zea mays mays*) oil is a rich source of polyunsaturated fatty acids (FAs) and energy, making it a valuable resource for human food, animal feed, and bio‐energy. Although this trait has been studied via conventional genome‐wide association study (GWAS), the single nucleotide polymorphism (SNP)‐trait associations generated by GWAS may miss the underlying associations when traits are based on many genes, each with small effects that can be overshadowed by genetic background and environmental variation. Detecting these SNPs statistically is also limited by the levels set for false discovery rate. A complementary pathways analysis that emphasizes the cumulative aspects of SNP‐trait associations, rather than just the significance of single SNPs, was performed to understand the balance of lipid metabolism, conversion, and catabolism in this study. This pathway analysis indicated that acyl‐lipid pathways, including biosynthesis of wax esters, sphingolipids, phospholipids and flavonoids, along with FA and triacylglycerol (TAG) biosynthesis, were important for increasing oil and FA content. The allelic variation found among the genes involved in many degradation pathways, and many biosynthesis pathways leading from FAs and carbon partitioning pathways, was critical for determining final FA content, changing FA ratios and, ultimately, to final oil content. The pathways and pathway networks identified in this study, and especially the acyl‐lipid associated pathways identified beyond what had been found with GWAS alone, provide a real opportunity to precisely and efficiently manipulate high‐oil maize genetic improvement.

## Significance Statement

This pathway analysis provided insights into the genetic basis of lipid metabolism, beyond what could be detected via GWAS alone, and indicated specific opportunities to precisely and efficiently manipulate high‐oil maize genetic improvement.

## Introduction

Oil content and composition are important determinants of maize (*Zea mays mays*) kernel quality (Watson, 1987). Maize oil is rich in high‐energy lipids in the form of triacylglycerols (TAGs), which include unsaturated fatty acids (FAs, e.g. oleic acid and linoleic acid), making maize oil a valuable resource for human food, animal feed, and bio‐energy. Different ratios of each FA may be more beneficial to different end uses. For instance, palm oil is well known for its high level of saturated FA and has many advantages for the food industry thanks to its high oxidative stability and high melting point, making it a good alternative to *trans*‐fats. Oils with higher oleic‐acid content are healthier because oleic acid can reduce blood pressure, inflammation, and oxidative damage, and may help prevent cancer. Therefore, understanding the genetic architecture of lipid metabolism in maize enables work on this key target for maize breeding and biotechnology‐assisted improvement. Maize kernel oil concentration and FA composition are highly heritable traits but are quantitative in nature and controlled by multiple genes.

Long‐term artificial selection in high‐oil maize populations has provided unique genetic resources for dissection of the genetic architecture of oil biosynthesis in maize kernels (Dudley and Lambert, 2004; Lambert *et al*., 2004; Song and Chen, 2004). In 1896, C. G. Hopkins started a selection experiment on the percent oil and protein of maize kernels using the open‐pollinated cultivar ‘Burr's White’ at the University of Illinois (Dudley and Lambert, 2004). He analyzed 163 ears for oil and protein content and selected the 24 ears highest in protein and another 24 for high oil, and the 12 ears lowest in protein and another 12 for low oil. Therefore, he initiated the Illinois High Oil (IHO), Illinois Low Oil (ILO), Illinois High Protein (IHP), and Illinois Low Protein (ILP) strains. After 100 generations of continued artificial selection, the kernel oil concentration of IHO increased from the initial 4.69 to 20.37%; in the ILO, after 85 generations it had decreased from 4.69 to 0.05% (below which germination was compromised; Dudley and Lambert, 2004). It is remarkable that 100 generations of selection had not eliminated the genetic variability in this population, and an upper limit has not been reached in the IHO (Dudley and Lambert, 2004). This indicates that many genes, each with a small effect, have been the targets of continual selection over the course of the experiment, and may continue to be so for some time to come.

Quantitative trait loci (QTL) for oil concentration and FA composition in maize have been identified from multiple studies of high‐oil maize lines (Sughrour and Rocheford, 1994; Alrefai *et al*., 1995; Laurie *et al*., 2004; Clark *et al*., 2006). These studies found several QTL with a range of small to large effects on different FA composition traits, many with additive effects. Using a recombinant inbred line (RIL) population, epistatic effects were found as well, and a gene under a QTL affecting maize seed oil and oleic‐acid content that encodes an acyl‐CoA:diacylglycerol acyltransferase (DGAT1‐2), which catalyzes the final step of oil synthesis, was identified by Yang *et al*. (2010). A phenylalanine insertion in *DGAT1‐2* increased oil and oleic‐acid content (Zheng *et al*., 2008). Our previous research combining linkage and association analyses allowed fine mapping of a QTL influencing levels of palmitic acid to a 90‐kb region containing a candidate gene, *Zea mays fatb* (*Zmfatb*), which encodes an acyl‐acyl carrier protein (ACP) thioesterase. An 11‐bp insertion in the last exon of this gene decreases palmitic acid content and concentration, leading to an optimization of the ratio of saturated to unsaturated FAs, while having no effect on total oil content (Li *et al*., 2011). Using near isogenic lines (NIL), Zhang *et al*. (2012) fine mapped a major QTL for embryo to endosperm ratio (EER) and kernel oil concentration, and identified *ZmGE2*, which encodes a cytochrome p450 protein. A 247‐bp transposable element (TE) insertion in the 3′‐untranslated region was associated with increased EER and kernel oil, and was a selection target during long‐term selection for high EER in a high‐oil population (Zhang *et al*., 2012).

Fine mapping with recombinant inbred lines (RILs) and map‐based cloning are highly accurate, but the process is time‐consuming and challenging. The genomic regions identified often span very large physical distances containing many repetitive sequences. Association mapping can shorten the research period while simultaneously analyzing greater allele numbers and greatly improving mapping resolution, often to the single gene level (Remington *et al*., 2001; Yu and Buckler, 2006). Beló *et al*. (2008) used GWAS to identify loci with major effects on oleic‐acid concentration in maize kernels and, with relatively few (8590) SNPs, identified *Zmfad2* as responsible for the differences in the oleic‐acid content. Recently, we conducted a GWAS with 1.03 million SNPs characterized in a panel of 368 maize inbred lines and identified 74 loci significantly associated with kernel oil concentration and FA composition, which we subsequently examined by expression QTL mapping, linkage mapping and coexpression analysis (Li *et al*., 2013). Most gene characterizations that have come from GWAS have discovered that the association is not actually in the causal gene; identifying the correct gene can be quite an arduous process.

Metabolic pathway analysis combined with GWAS focuses on the cumulative effects of many genes grouped according to their shared biological function. This promising approach can give clues to the genetic basis of a trait by finding biological insights missed when focusing on only one or a few genes that are most significantly associated (Tang *et al*., 2015). Although originally developed to study differences in gene expression data for medically important diseases (Subramanian *et al*., 2005), pathway analysis has also been used with association mapping data, first in human disease studies (Wang *et al*., 2007) and then plants (Tang *et al*., 2015). In addition, biologically relevant pathways can be used to determine additional candidate genes for further study or to interpret large data sets produced by other high‐throughput approaches, including RNA sequencing, proteomics, and metabolomics. This is being done in maize, in targeted pathways such as carotenoid biosynthesis (Owens *et al*., 2014); in functionally related networks (Baute *et al*., 2016; Walley *et al*., 2016); and in protein interaction networks (Musungu *et al*., 2015).

Tang *et al*. (2015) took the pathway‐based approach using the results of an aflatoxin GWAS study in maize to jointly consider all genetic sequences associated with aflatoxin accumulation resistance, regardless of magnitude of allele effects. GWAS alone identified significant associations but none led to plausible resistance mechanisms; 17 high ranking pathways representing mechanisms to prevent fungal growth and production of deleterious aflatoxin were identified via the pathway approach (Tang *et al*., 2015). Many of these pathways were consistent with known resistance mechanisms, primarily centered on the production of the bioactive plant hormone methyl‐jasmonate, known to orchestrate plant responses to several biotic stresses. The interaction of production pathways of different lipids makes it difficult to find genes that account for all phenotypic variation of one FA alone. Levels of one FA are generally linked to levels of others, as production of one may start with, and therefore decrease, another (Li‐Beisson *et al*., 2013). To better understand lipid metabolism in maize and identify key points for genetic or genomic manipulation, this study was performed to elucidate the biochemical pathways, metabolic networks, and associated genes that contribute to oil‐related traits by accounting for linkage disequilibrium and association statistics among SNPs from a large‐scale GWAS study.

## Results

### GWAS and tagSNPs

SNP‐trait associations calculated for oil concentration and 9 FA‐related traits were reported in Li *et al*. (2013) and Table S1. The GWAS was run with TASSEL on 560 000 SNPs genotyped on each of the 368 inbred maize lines in this study. In total, 139 SNPs associated at *P *<* *1.8 × 10^−6^ were identified for oil concentration, for which *R* ^2^ values ranged between 6.61 × 10^−6^ to 0.18 (Li *et al*., 2013). A pathway analysis was run using the cumulative association information from all SNPs in the GWAS, by identifying tagSNPs and linked genes per linkage block and assigning them to common metabolic pathways. In total, 259 206 unique tagSNPs were used to locate 25 404 genes, of which 3106 mapped to the 313 MaizeCyc pathways that had five or more genes. This information, along with the number of tagSNPs and genes mapped to the significant pathways, is shown in Table S2.

### Most significant pathways for increasing oil concentration

**Figure 1:**
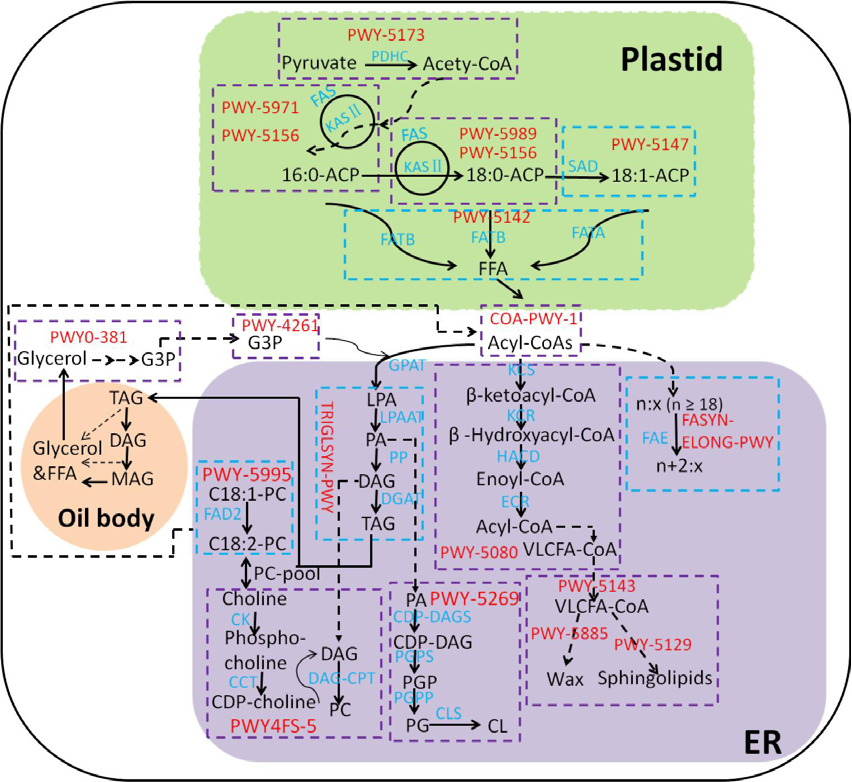
The simplified lipid metabolic pathway in maize. The reactions corresponding to significant pathways detected by pathway analysis are in purple and blue dotted line boxes. The colored boxes also denote pathways that were missed (purple) and detected (blue) by GWAS alone (Li *et al*., 2013). Plastid and endoplasmic reticulum (ER) are shaded with green and purple, respectively. PDHC, pyruvate dehydrogenase complex; FAS, fatty acid synthase; KAS, ketoacyl‐ACP synthase; SAD, stearoyl‐ACP desaturase; ACP, acyl carrier protein; FAT, acyl‐ACP thioesterase; FFA, free fatty acid; FAE, fatty acid elongase. KCS, ketoacyl‐CoA synthase; KCR, ketoacyl‐CoA reductase; HACD, hydroxyacyl‐CoA dehydrase; ECR, enoyl‐CoA reductase; VLCFA, very long‐chain fatty acid; G3P, glycerol 3‐phosphate; GPAT, glycerol 3‐phosphate acyltransferase; LPA, lysophosphatidic acid; PA, phosphatidic acid; LPAAT, lysophosphatidic acid acyltransferase; DAG, diacylglycerol; PP, PA phosphatase; TAG, triacylglycerol; DGAT, acyl‐CoA: diacylglycerol acyltransferase; MAG, monoacylglycerol; FAD2, oleate desaturase; CK, choline kinase; CCT, choline‐phosphate cytidylyltransferase; DAG‐CPT, diacylglycerol cholinephosphotransferase; PC, phosphatidylcholine; CDP‐DAG, CDP‐diacylglycerol; CDP‐DAGS, CDP‐DAG synthase; PGPS, phosphatidylglycerophosphate synthase; PGP, phosphatidylglycerol phosphate; PGPP, PGP phosphatase; PG, phosphatidylglycerol; CL, cardiolipin; CLS, cardiolipin synthase. Pathway names: PWY‐5173, superpathway of acetyl‐CoA biosynthesis; PWY‐5971, palmitate biosynthesis II (bacteria and plants); PWY‐5156, superpathway of fatty acid biosynthesis II (plant); PWY‐5989, stearate biosynthesis II (plants); PWY‐5147, oleate biosynthesis I (plants); PWY‐5142, acyl‐ACP thioesterase pathway; COA‐PWY‐1, coenzyme A biosynthesis; PWY‐4261, glycerol degradation IV; PWY‐5080, very long‐chain fatty acid biosynthesis; PWY‐5143, fatty acid activation; PWY‐5885, wax esters biosynthesis II; PWY‐5129, sphingolipid biosynthesis (plants); TRIGLSYN‐PWY, triacylglycerol biosynthesis; FASYN‐ELONG‐PWY, fatty acid elongation – saturated; PWY0‐381, glycerol degradation I; PWY‐5995, linoleate biosynthesis I (plants); PWY4FS‐5, superpathway of phosphatidylcholine biosynthesis; PWY‐5269, cardiolipin biosynthesis II.

**Figure 2:**
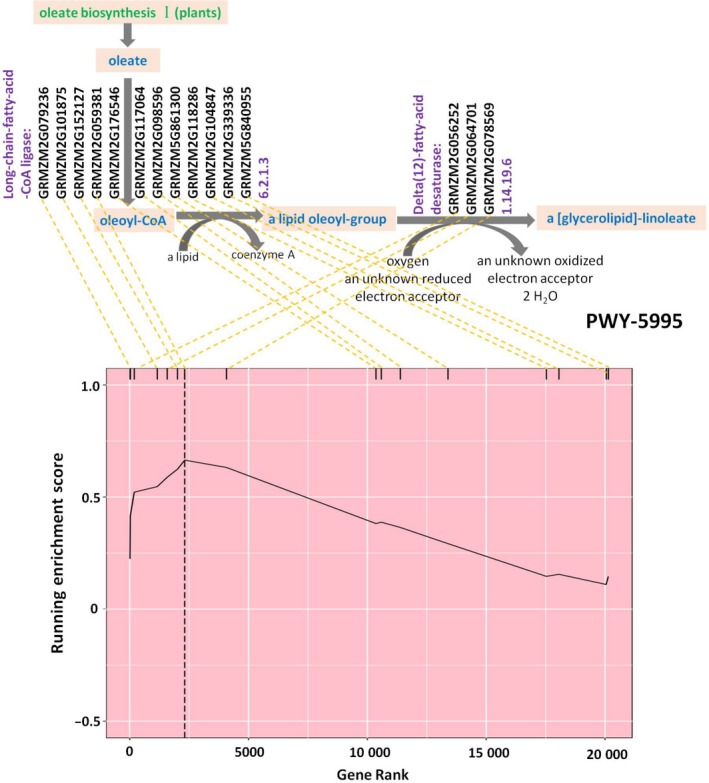
Graphs of pathway reactions and the running enrichment score (RES) calculation for PWY‐5995, linoleate biosynthesis I. Genes were ranked in descending order (left to right) by their effect scores and paired using the yellow dotted line with genes in the pathway reaction, denoted by hash marks at the top of the RES graph. The pathway enrichment score that coincided with the maximum running enrichment score is marked by the black vertical dashed line.

Three pathways were significantly associated with increased oil concentration at *P *<* *0.01, and an additional 16 at *P *<* *0.05 (Table 1). These include seven pathways directly involved in lipid metabolism (Table 1), and some of these are shown in Figure 1. Linoleate biosynthesis I (PWY‐5995) was the most significant (*P *=* *3.51 × 10^−4^) pathway identified in this study. Linoleate is a polyunsaturated FA that accounts for more than 50% of the oil content in the maize grains of this panel (Li *et al*., 2013). Most of the oleoyl‐CoA converted from oleoyl‐ACP is incorporated into lipids, forming phosphatidylglycerols (PGs), diglycerides, and phosphatidylcholines (PCs). Further desaturation of the oleoyl groups to linoleoyl groups in the endoplasmic reticulum (ER) occurs while being incorporated into lipids (Figure 2). This step is catalyzed by the enzyme acyl‐lipid ω‐6 desaturase (EC1.14.19.f, or cytochrome b5), encoded by the *FAD2* gene, which was also significantly associated with oil concentration in our previous GWAS results (Li *et al*., 2013). This gene was the second most important in the calculation of the running enrichment score (RES) for PWY‐5995; all genes in the pathway can be seen in Figure 2, along with their relative contribution to oil concentration (genes are sorted and denoted by the hash marks along the top of the RES graph). The most important gene in the pathway (according to enrichment score), which had long‐chain fatty acid‐CoA ligase activity (MaizeCyc v2.1; Monaco *et al*., 2013), was not identified in the GWAS by Li *et al*. (2013).

**Table 1:** Pathways associated with increased oil concentration and with enrichment scores that were significant at *P *<* *0.05. Pathway identifier (ID) and name are drawn from the MaizeCyc database (https://www.maizegdb.org/metabolic_pathways/), and *P* and FDR were calculated in this study

MaizeCyc ID	Pathway name	*P*	FDR
PWY‐5995	Linoleate biosynthesis I (plants)	0.00035	0.10553
PWY‐5121	Superpathway of geranylgeranyldiphosphate biosynthesis II (via MEP)	0.00273	0.40943
PWY‐5143	Fatty acid activation	0.00684	0.61996
PWY‐5912	2′‐Deoxymugineic acid phytosiderophore biosynthesis	0.01065	0.61996
PWY‐5687	Pyrimidine ribonucleotides interconversion	0.01489	0.61996
NONMEVIPP‐PWY	Methylerythritol phosphate pathway	0.01522	0.61996
PWY‐5123	*Trans*,* trans*‐farnesyl diphosphate biosynthesis	0.01543	0.61996
PWY‐4081	Glutathione redox reactions I	0.01879	0.61996
PWY0‐163	Salvage pathways of pyrimidine ribonucleotides	0.02114	0.61996
PWY‐5366	Palmitoleate biosynthesis II	0.02588	0.61996
PWY‐5142	Acyl‐ACP thioesterase pathway	0.02588	0.61996
UDPNACETYLGALSYN‐PWY	UDP‐*N*‐acetyl‐D‐glucosamine biosynthesis II	0.02665	0.61996
TRIGLSYN‐PWY	Triacylglycerol biosynthesis	0.02689	0.61996
PWY2OL‐4	Linalool biosynthesis	0.03074	0.61996
SO4ASSIM‐PWY	Sulfate reduction I (assimilatory)	0.03532	0.61996
PWY‐5340	Sulfate activation for sulfonation	0.03532	0.61996
COA‐PWY‐1	Coenzyme A biosynthesis	0.04194	0.61996
PWY‐5885	Wax esters biosynthesis II	0.04270	0.61996
PWY‐5278	Sulfite oxidation III	0.04559	0.61996

Another pathway with enrichment scores significant at the *P *<* *0.05 is the TAG biosynthesis pathway (TRIGLSYN‐PWY, Figure 1 and Table 1). The major lipid in maize grains reserve is TAG, a glycerol backbone onto which three FAs are sequentially esterified in higher plants. The assembly of TAG occurs in the ER by four consecutive reactions. *DGAT*, encoding diacylglycerol acyltransferase, catalyzes the final step of TAG synthesis, which was associated with oil concentration in our previous GWAS result (Li *et al*., 2013). Over‐expression of *DGAT* increases oil and oleic‐acid concentration by up to 41 and 107% in maize kernels, respectively (Zheng *et al*., 2008).

### Most significant pathways for increasing FA

Pathway analyses were run on associations calculated for nine FA concentration traits, including the concentration levels of palmitic (C16:0), stearic (C18:0), oleic (C18:1), linoleic (C18:2), and linolenic (C18:3) acids, and the ratios of palmitic to stearic (C16:0/C18:0), stearic to oleic (C18:0/C18:1), oleic to linoleic (C18:1/C18:2), and linoleic to linolenic (C18:2/C18:3). Of the 313 MaizeCyc pathways analyzed, 28 that increased the concentration of one or more FA were identified, and had enrichment scores that were significant at *P *<* *0.01 (Table 2). Of these, 10 are directly involved in lipid metabolism, including known biosynthesis pathways for palmitate, stearate, oleate, and linoleate (Li‐Beisson *et al*., 2010). An extended list for pathways with enrichment scores significant at *P *<* *0.05 is included in Table S3. In addition to the direct biosynthesis pathways, others that are known to be involved in lipid metabolism were identified (Figure 1). Pathway PWY‐5147, oleate biosynthesis I, identified at *P < *0.01 in two traits, is central to lipid metabolism and has two sequential reactions that begin with stearoyl‐ACP. Based on the ranks of the effect values, 15 of the 19 genes in this pathway contributed to the enrichment score (Table S4). Two of these genes (*GRMZM5G829544* and *GRMZM2G079308*) had also been identified via GWAS (Li *et al*., 2013). The key enzyme in this pathway is stearoyl‐ACP 9‐desaturase (SAD), which introduces the first double bond at the delta (9) position of stearoyl‐ACP to produce oleoyl‐ACP. This enzyme is responsible for the conversion of saturated FAs to unsaturated FAs in the synthesis of vegetable oils (Dyer and Mullen, 2001; Han *et al*., 2017). This example, and all pathway results, illustrates the effectiveness of the pathway analysis for interpreting GWAS results beyond what is seen simply with single point association data (Tables S1 and S4).

**Table 2:** Pathways associated with increased FA concentration and with enrichment scores that were significant at *P *<* *0.01. Pathway identifier (ID) and name are drawn from the MaizeCyc database (https://www.maizegdb.org/metabolic_pathways/), and *P* and FDR were calculated in this study

MaizeCyc ID	Lead trait^a^	Other trait^b^	Pathway name	Lead trait *P‐*value	Lead trait FDR	Other trait FDR
COA‐PWY‐1	C16:0		Coenzyme A biosynthesis	0.00399	0.15680	
PWY‐5035	C16:0		Gibberellin biosynthesis III (early C‐13 hydroxylation)	0.00585	0.18314	
PWY‐5147	C16:0	C18:1	Oleate biosynthesis I (plants)	0.00138	0.15680	0.22442
PWY‐5995	C16:0		Linoleate biosynthesis I (plants)	0.00378	0.15680	
FASYN‐ELONG‐PWY	C18:0		Fatty acid elongation – saturated	0.00364	0.15615	
PWY‐5142	C18:0		Acyl‐ACP thioesterase pathway	0.00449	0.15615	
PWY‐5156	C18:0	C16:0	Superpathway of fatty acid biosynthesis II (plant)	0.00146	0.07626	0.15680
PWY‐5366	C18:0		Palmitoleate biosynthesis II	0.00449	0.15615	
PWY‐5367	C18:0	C16:0	Petroselinate biosynthesis	0.00009	0.00926	0.15680
PWY‐5971	C18:0	C16:0	Palmitate biosynthesis II (bacteria and plants)	0.00012	0.00926	0.15680
PWY‐5973	C18:0	C16:0	*Cis*‐vaccenate biosynthesis	0.00009	0.00926	0.15680
PWY‐5989	C18:0	C16:0	Stearate biosynthesis II (plants)	0.00012	0.00926	0.15680
PWY‐6151	C18:0		*S*‐adenosyl‐l‐methionine cycle I	0.00113	0.07093	
ARO‐PWY	C18:1		Chorismate biosynthesis I	0.00112	0.17456	
PWY‐4081	C18:1		Glutathione redox reactions I	0.00904	0.41883	
PWY‐5912	C18:1	C16:0	2′‐Deoxymugineic acid phytosiderophore biosynthesis	0.00306	0.22442	0.18314
PWY‐6457	C18:1		*Trans*‐cinnamoyl‐CoA biosynthesis	0.00937	0.41883	
PWY‐6628	C18:1		Superpathway of phenylalanine biosynthesis	0.00200	0.20860	
PWY‐6629	C18:1		Superpathway of tryptophan biosynthesis	0.00098	0.17456	
PWY‐5340	C18:2		Sulfate activation for sulfonation	0.00462	0.48231	
PWY‐5687	C18:2		Pyrimidine ribonucleotides interconversion	0.00693	0.54214	
SO4ASSIM‐PWY	C18:2		Sulfate reduction I (assimilatory)	0.00462	0.48231	
UDPNACETYLGALSYN‐PWY	C18:2		UDP‐*N*‐acetyl‐d‐glucosamine biosynthesis II	0.00322	0.48231	
PWY1F‐353	C18:3		Glycine betaine biosynthesis III (plants)	0.00469	0.36720	
PWY‐3282	C18:3		Ammonia assimilation cycle II	0.00125	0.36720	
PWY‐381	C18:3		Nitrate reduction II (assimilatory)	0.00426	0.36720	
PWY‐5934	C18:3		Fe(III)‐reduction and Fe(II) transport	0.00594	0.37199	
PYRIDNUCSYN‐PWY‐1	C18:3		NAD biosynthesis I (from aspartate, plastidic)	0.00314	0.36720	

a The pathways with the most significant *P*‐value among FA concentration traits.

b Additional FA concentration trait with significant *P*‐value (*P *<* *0.01).

The identified pathways often influenced more than one of the FAs, which was expected because FAs are frequently modified or converted from one into another (Tables 2 and S3). The correlation between the levels of all FAs is strongly significant (*P *<* *0.001, Li *et al*., 2013), and the same pathways are frequently identified when running the analysis on different FAs at *P *<* *0.05 (Tables 2 and S3). In addition, single pathways often affect the levels of multiple FAs. For example, the coenzyme A biosynthesis pathway (COA‐PWY‐1) had a high enrichment score for C16:0 and C18:2; FA elongation – saturated (FASYN‐ELONG‐PWY) had a high enrichment score for C16:0 and C18:0; and the acyl‐ACP thioesterase pathway (PWY‐5142) had a high enrichment score for C16:0, C18:0 and C18:1 (Figure 1; Tables 2 and S3).

**Figure 3:**
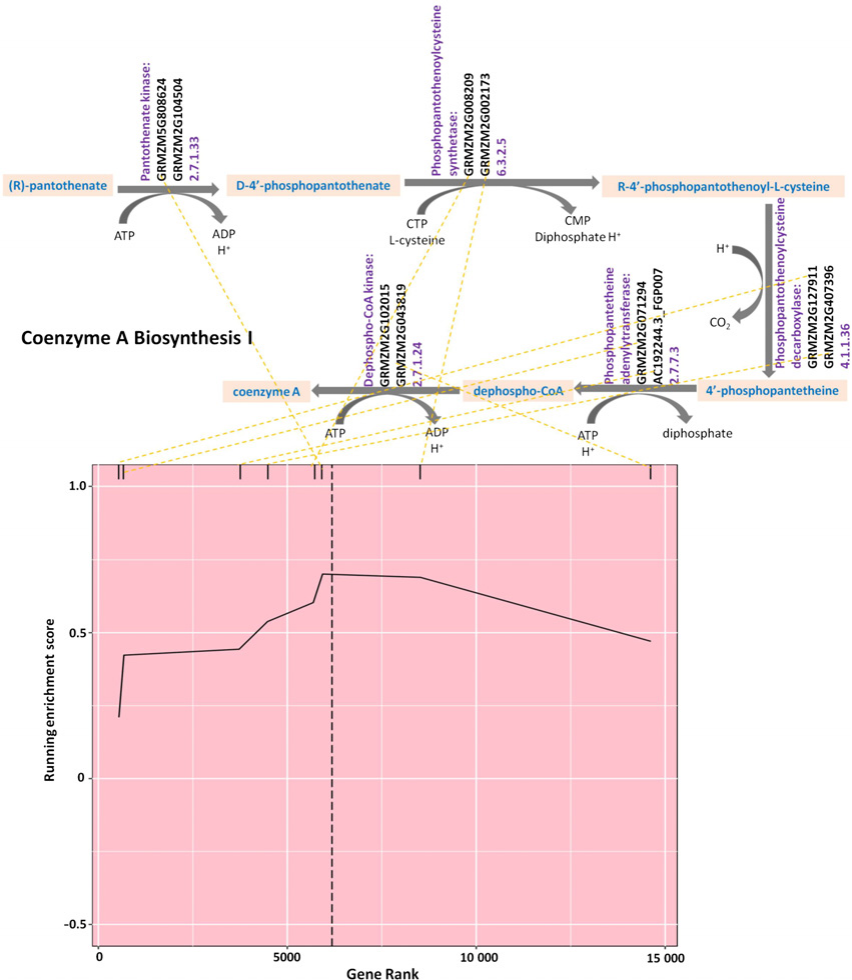
Graphs of pathway reactions and the running enrichment score (RES) calculation for COA‐PWY‐1, coenzyme A biosynthesis. Genes were ranked in descending order (left to right) by their effect scores and paired using the yellow dotted line with genes in the pathway reaction, denoted by hash marks at the top of the RES graph. The pathway enrichment score that coincided with the maximum running enrichment score is marked by the black vertical line.

Coenzyme A (CoA) functions as an acyl carrier and carbonyl‐activating group in numerous central biochemical transformations, including the tricarboxylic acid cycle (TCA, also known as the citric acid cycle (CAC) or Krebs cycle) and FA metabolism (Rubio *et al*., 2006). The biosynthesis of CoA from (R)‐pantothenate (Figure 3) is an essential universal pathway in both prokaryotes and eukaryotes. In the present analysis, eight genes in the CoA biosynthesis pathway (COA‐PWY‐1) contributed the most to the enrichment score. The steps in the pathway mediated by the products of these genes are indicated in Figure 3 as well. Therefore, it can be seen where the allelic variation in this panel was critical in the biosynthesis of CoA for determining the level of C16:0 and C18:2 in maize kernels. The FA elongation pathway (FASYN‐ELONG‐PWY) includes the reactions that constitute one turn of a cycle that lengthens the chain of an acyl‐ACP molecule by two carbons. The products of this pathway are saturated FAs such as C12:0, C14:0, C16:0 and C18:0 (Li‐Beisson *et al*., 2010), and the importance of this pathway was confirmed by our results (Table S3). None of the genes in these two pathways was identified in the GWAS results of Li *et al*. (2013). In this study's analysis, however, the cumulative effect of many genes in the pathway allowed it to be identified as associated with multiple measured traits. The pathway for acyl‐ACP thioesterase (PWY‐5142) is encoded by nine genes, and releases FA from acyl‐ACPs. Two of these nine genes, *GRMZM2G079308* (*FATB.a*) and *GRMZM5G829544* (*FATB.b*), contributed to increasing the RES. These two genes were identified in previous GWAS results (Li *et al*., 2013; Table S1). The main products of acyl‐ACP thioesterase B (FATB) are C16:0 and to a lesser extent C18:0 in all plants (Salas and Ohlrogge, 2002; Li‐Beisson *et al*., 2013).

In addition to FA biosynthesis pathways, acyl‐lipid pathways were also identified in this analysis of increased FA traits. These included wax esters biosynthesis II (PWY‐5885) for C18:1; sphingolipid biosynthesis (PWY‐5129) and phospholipid biosynthesis II (PHOSLIPSYN2‐PWY) for C18:2; flavonoid biosynthesis (PWY1F‐FLAVSYN) for C18:2 and C18:3; and the superpathway of phosphatidylcholine biosynthesis (PWY4FS‐5) for C18:3. The wax esters biosynthesis pathway (PWY‐5885) is carried out by several unrelated acyltransferases, including wax ester synthase (WS)/DGAT. Wax esters biosynthesis uses very long‐chain fatty acids (VLCFA) (elongated from small to medium length FAs), which are esterified to a short chain alcohol (King *et al*., 2007). Flavonoids are secondary metabolites formed from phenylpropanoid and FA derivatives and have an important function, acting as UV‐B protectors, signal molecules in legume−rhizobium bacteria interactions, and in response to biotic stress (Peters *et al*., 1986; Ryan *et al*., 2001). Pathways involved in hormone synthesis, amino acid degradation, and energy production also contributed to the increase in FA levels in this analysis (Figure 1; Tables 2 and S3).

### Most significant pathways for decreasing oil concentration and FA

**Figure 4:**
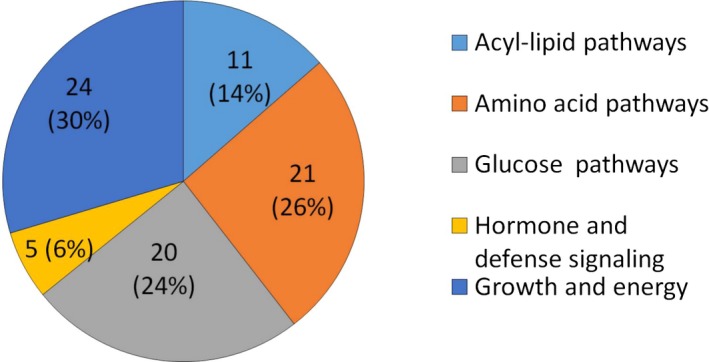
Pathway annotation category for the 86 significantly associated pathways for decreasing oil content and FA.

The pathways associated with a decrease in oil and FA concentration can be found in Table S5. Among the 86 significantly (*P *<* *0.05) associated pathways, 11 were related to lipid metabolism. These were mainly lipid degradation pathways, and 23 were related to degradation of primary metabolites in general (Figure 4; Table S5). For instance, glycerol degradation IV (PWY‐4261) was the most significantly enriched in the analysis of C18:0 (*P *<* *0.01) and C18:1 (*P *<* *0.05, Figure 1; Table S5). During seed germination, TAG is broken down to FAs and glycerol, which are converted to sucrose via the glyoxylate cycle to support seedling growth (Li‐Beisson *et al*., 2013). In the glycerol degradation pathway (PWY‐4261), glycerol is phosphorylated to glycerol‐3‐phosphate by glycerol kinase, and then converted to dihydroxyacetone phosphate by glycerol‐3‐phosphate dehydrogenase, *acs7*. The product of glycerol degradation, dihydroxyacetone phosphate, can be converted to sugars via gluconeogenesis (Friso *et al*., 2010). Therefore, there may be less glycerol‐3‐phosphate available for TAG biosynthesis and subsequent production of oil. The *acs7* gene, as well as 12 others, together increased the enrichment score of PWY‐4261; these genes were not identified in the GWAS results of Li *et al*. (2013).

Another pathway reducing lipids is heptadecane biosynthesis (PWY‐6622), which was found to be enriched in the analysis of C16:0 and C18:0 (*P *<* *0.05, Table S5). The alkanes are produced from FAs in three steps – activation of the FA by an ACP, reduction of the activated FA to an aldehyde, and decarbonylation of the aldehyde by *gl1*, resulting in an alkane that is one carbon shorter than the original FAs. Therefore, the increase in metabolites derived from FAs would decrease the level of FAs, and this was shown in our results. The list of pathways that decrease oil and FA concentration was dominated by carbon partitioning pathways, such as amino acid and glucose related pathways, which made up over 50% of total pathways with decreasing effect values (Figure 4; Table S5). Examples of significant substrate consumption pathways include pyruvate fermentation to ethanol (PWY‐5486) and gluconeogenesis I (GLUCONEO‐PWY) (Table S5). Gluconeogenesis is the generation of glucose from non‐sugar carbon substrates such as pyruvate and glycerol. The process is essentially the reversal of the glycolysis pathway. In addition to the important role played by carbon partitioning in lipid metabolism, pathways involved in hormone production, cell growth, or energy production decrease lipid levels as well (Figure 4).

## Discussion

The candidate genes identified by previous GWAS of oil‐related traits were mostly involved in FA and TAG biosynthesis (Li *et al*., 2013; Table S1). Following biosynthesis, FAs can then be subjected to elongation, desaturation and eventual export from the plastid, ultimately giving rise to distinct acyl‐lipid species, including phosphatidylinositols (PIs), phosphatidylcholines (PCs), phosphatidylethanolamines (PEs), phosphatidylglycerols (PGs), monogalactosyldiacylglycerols (MGDGs), digalactosyldiacylglycerols (DGDGs), sulfoquinovosyldiacylglycerols (SQDGs) (Li‐Beisson *et al*., 2013). However, the large number of intermediates and the interaction between lipid pathways render the characterization of lipid metabolism quite challenging (De Abreu *et al*., 2018). Pathway analysis of oil‐related traits provided a complementary method to detect the mechanisms of lipid metabolism in maize kernels and emphasized multiple aspects of SNP‐trait associations rather than just significance, as in conventional GWAS.

Our pathway analysis results indicate that acyl‐lipid pathways, including wax esters biosynthesis, sphingolipid biosynthesis, phospholipid biosynthesis and flavonoid biosynthesis, were as important as FA and TAG biosynthesis pathways for increasing oil and FA concentration (Tables 1, S1 and S3), and for changing FA ratios (Tables S6). Due to the nature of statistical tests, it is possible that one or more of the pathways identified by this analysis was a false positive result, and not actually involved in oil biosynthesis. However, resampling with replacement and false discovery rate protection were both done to reduce the incidence of false positives. It is therefore likely that detected pathways which are not known lipid biosynthesis pathways do contribute to overall levels of lipids in maize kernels, in a less direct manner. Further experiments with molecular biology tools and/or CRISPR/Cas9 would be required to provide validation of involvement of all pathways in oil biosynthesis.

### Lipid metabolism in the maize kernel

By combining pathway analysis results with knowledge about lipid metabolism in the model species Arabidopsis (Li‐Beisson *et al*., 2013), we can propose further modifications that can be used to achieve optimum levels of FAs and lipids in maize for different end uses. The acyl‐lipid metabolic pathways in maize (Figure 1) and Arabidopsis generate an acetyl‐CoA pool from pyruvate through the action of the plastidial pyruvate dehydrogenase complex (PDHC). FAs are synthesized in the plastid by a Type II fatty acid synthase complex (FAS) adding two carbon units to the extending FA chain in serial reactions. Resulting FAs are typically 16 or 18 carbons long and attached to an ACP. Stearic acid (18:0‐ACP) can be desaturated by SAD. Long‐chain acyl groups are then hydrolyzed by FAT that release FAs, which are ultimately activated to CoA esters and exported to the ER. In the ER, they are assembled into phosphatidic acid (PA) in the acyl‐CoA‐dependent Kennedy pathway with the enzymes glycerol‐3‐phosphate acyltransferases (GPATs) and lysophosphatidic acid acyltransferases (LPAATs), and acyl‐CoA. This PA can then be dephosphorylated *de novo* by PA phosphatases (PP) to create diacylglycerol (DAG), which is then available for acyltransferase reaction. DGAT transfers acyl‐CoAs to the sn‐3 position of DAG to produce TAG. Phosphatidylcholine (PC) is created via the CDP‐choline pathway using CDP‐choline:diacylglycerol cholinephosphotransferase (DAG‐CPT); and cardiolipin (CL) is created via the action of enzymes phosphatidylglycerol phosphate (PGP) synthase (PGPS), PGP phosphatase (PGPP), and CL synthase (CLS).

The first step in wax and sphingolipid biosynthesis is an elongation cycle converting C16:0 and C18:0 fatty acyl‐CoAs to generate VLCFA wax and sphingolipid precursors between 20 and 34 carbons in length. The first step involves condensation of malonyl‐CoA with an acyl‐CoA catalyzed by a β‐ketoacyl‐CoA synthase (KCS). The resulting β‐ketoacyl‐CoA is reduced by a β–ketoacyl‐CoA reductase (KCR) and the resulting β‐hydroxyacyl‐CoA then undergoes dehydration by a β–hydroxyacyl‐CoA dehydratase (HCD). In the final step, the enoyl‐CoA is reduced to an acyl‐CoA by enoyl‐CoA reductase (ECR). This cycle results in an acyl chain extended by two carbons, and the cycle can be repeated. FA elongation is catalyzed by an ER‐associated, multienzyme complex known as fatty acid elongase (FAE). Therefore, the levels of specific FAs, and total oil, depend on the synthesis of these lipids from precursors, which are frequently other lipids, and the degradation of these lipids (often into other lipids, but also many other compounds). There will be many more pathways associated with the amount of each FA, ratios of FAs, and total oil than simply the biosynthesis pathways that create them, and these pathways will be a key to achieve target levels of any specific FA or lipid.

### Practical modification of lipid metabolism guided by pathway analysis

The GWAS by Li *et al*. (2013) identified 74 loci significantly associated with kernel oil concentration and fatty acid composition. Many of these genes are in pathways that were identified in the current study (Table S1). However, many of those genes are not in pathways in the MaizeCyc database, so they could not be identified by pathway analysis. Conversely, many of the pathways identified in the current study contained no genes identified by GWAS, as no single gene was associated at significance levels exceeding the GWAS threshold, but the cumulative RES of multiple genes in these pathways allowed them to be identified. We therefore see that the two analyses are complementary for identifying more mechanisms and candidate genes for complex traits.

We have shown that identification of biosynthesis pathways for FA derivatives greatly broaden understanding of mechanisms of lipid metabolism and/or catabolism. Because starch, oil and protein are the three main components in maize kernels, the ability to manipulate oil and FA concentrations now allows breeders to create specialty maize for different breeding targets. There is a tradeoff between the lipid and starch components in maize grain (Schwender *et al*., 2015). Increasing lipid content also increases plastidic fatty acid synthesis and glycolytic flux, while decreasing glycolytic intermediates. In addition, the lipid/starch tradeoff is most likely mediated by allosteric feedback regulation of phosphofructokinase and ADP‐glucose pyrophosphorylase. Our pathway results provide hints as to the mechanism of this lipid/starch tradeoff. Allelic variation exists within the genotypes of this GWAS panel that should allow precise manipulation of FA content within the FA biosynthesis and carbon partitioning pathways. Genes at critical branching points of pathways with the strongest associations to the desired FA can be targeted via allele mining or genome editing to create lines with the exact desired levels of these FA.

TAG is the predominant form of storage lipids in seeds of oil crops. It is clear from this pathway analysis that the synthesis and assembly of TAG in plants involves a metabolic network of FA fluxes through multiple subcellular compartments containing alternative pathways to produce different lipid compositions. Therefore, systems biology offers a powerful and effective tool to assemble multiple genes involved in lipid metabolic engineering. For example, genes for competing pathways can be disrupted to direct metabolic flux toward a desired route. From Figure 1, we see that *FAD2* encodes the enzyme that converts 18:1 into 18:2. Genome editing could be used to ‘knock out’ or reduce FAD2 activity for enhancement of oleic acid. This approach has been demonstrated by silencing the soybean *FAD2* gene family to create a high oleic‐acid seed trait (Haun *et al*., 2014). In addition, *DGAT1‐2* is responsible for the TAG content (Figure 1). We could reduce FAD2 activity and increase DGAT1‐2 activity for enhancement of oleic acid and TAG simultaneously to design a maize line with high oleic acid and oil content.

While yield improvement has long been the primary goal of conventional maize breeding, other nutritional or industrial traits are becoming more important. This pathway analysis of lipid metabolism provides information to identify which pathways should be tweaked at one or a few key genes each, to create lines with the precise levels or ratios of lipids desired. This information may soon be used, along with the development and application of synthetic biology tools to design and assemble large DNA constructs, and/or gene editing, to bring target lipid synthesis traits together with excellent agronomic and yield performance in the creation of superior high‐oil maize lines.

## Experimental procedures

### GWAS dataset

The GWAS analysis results of Li *et al*. (2013), which were used as input data for the pathway analysis are described briefly as follows. A GWAS analysis was run on a panel of 368 diverse inbred lines including 23 high‐oil lines. All entries were genotyped using RNA sequencing. About 560 000 polymorphisms with minor allele frequency (MAF) ≥ 0.05 were selected for analysis. Linkage disequilibrium (LD) was calculated between each pair of SNPs in TASSEL (Bradbury *et al*., 2007). Overall LD decay was rapid, reaching 500 bp (*r* ^2^ = 0.1) in the 368 lines.

All 368 lines were phenotyped for oil concentration and composition in replicated field experiments in four environments in China. Levels of total oil and 10 individual FAs in the kernels were measured according to Yang *et al*. (2010) and reported in Li *et al*. (2013), along with ratios of different FAs. The pathway analyses presented here were run on oil concentration, the FA concentration of palmitic (C16:0), stearic (C18:0), oleic (C18:1), linoleic (C18:2), and linolenic (C18:3) acids, and the ratios of palmitic to stearic (C16:0/C18:0), stearic to oleic (C18:0/C18:1), oleic to linoleic (C18:1/C18:2), and linoleic to linolenic (C18:2/C18:3), 10 traits in all.

### Pathway analysis

The resulting SNP‐trait association data generated by TASSEL (Bradbury *et al*., 2007) were implemented in the pathway analysis according to Tang *et al*. (2015) and included the SNP‐trait association values for significance (*P*)*,* correlation (*R* ^2^ or proportion of the phenotypic variation accounted for), and effect values along with the calculated LD values for *D*′*,* and *R* ^2^, and *P* between each marker SNP and its closest neighboring SNPs (50 upstream and 50 downstream). SNPs were then assigned to genes using a tagSNP approach outlined by Tang *et al*. (2015) in their decision tree. Briefly, if LD analysis showed no linkage between a reference SNP and other SNPs, then the reference SNP was the tagSNP. If LD analysis found linkage between the reference SNP and a block of other SNPs in either upstream and downstream directions, then selection of the tagSNP from the reference SNP or the SNPs in the linkage block was based on the SNP‐trait association effect values (majority sign, positive or negative, and magnitude), *P‐*values, and distance between the reference SNP and closest SNP in the linkage block. The purpose of the tagSNP was to reduce the dimensionality of the dataset to SNPs with the greatest effects on the trait under analysis. The gene(s) causing the SNP‐trait association was then assumed to be within 1 Kb of the SNP. The reason for using only 1 kb is because many *cis*‐acting elements that regulate gene expression reside within the gene or in immediate regions upstream and downstream of the gene coding region. If a gene was located, then the SNP‐trait association effect value was transferred to the gene and used for gene‐set enrichment analysis (Tang *et al*., 2015). Gene sets were defined as the genes according to membership in pathways and superpathways found in the MaizeCyc database (MaizeCyc v2.1; Monaco *et al*., 2013). Only pathways with five or more genes (313 pathways in total) were considered to reduce bias from a small sample size. Genes were ranked by their effects (negative to positive), and a running sum statistics similar to a weighted Kolmogorov−Smirnov statistics was calculated. The weighting factor for the running sum statistics was the absolute value of the SNP‐trait association effect. The enrichment score for the pathway was the maximum positive deviation of the running sum statistics from zero. Significance of the enrichment score was determined by permutation analysis (1000 random permutations of the effect values). The *P‐*values for the pathway enrichment scores were then corrected for false discovery rate (FDR) using the QVALUE package in R (Storey and Tibshirani, 2003, R package version 2.2.2. http://github.com/jdstorey/qvalue).

## Conflict of Interest

The use of trade name, commercial product or corporation in this publication is for the information and convenience of the reader and does not imply an official recommendation, endorsement or approval by the US Department of Agriculture or the Agricultural Research Service for any product or service to the exclusion of others that may be suitable. USDA is an equal opportunity provider and employer. The authors declare that they have no competing interests.

## Supporting information


**Table S1.** Summary of significantly associated genes from the GWAS study of Li *et al*. (2013), and those that were also identified by pathway analysis. Pathway identifier (ID) and name are drawn from the MaizeCyc database (https://www.maizegdb.org/metabolic_pathways/).
**Table S2.** Summary of significantly associated SNPs from the GWAS study of Li *et al*. (2013) with *R* ^2^ values, and the number of tagSNPs and genes from the present study.
**Table S3.** Pathways associated with increased FA content and with enrichment scores that were significant at 0.01 < *P *<* *0.05. Pathway identifier (ID) and name are drawn from the MaizeCyc database (https://www.maizegdb.org/metabolic_pathways/), and *P* and FDR were calculated in this study.
**Table S4.** Number of genes which contributed to the enrichment score in significant pathways at *P *<* *0.01, and which are shown as hatch marks at the top of the running enrichment score graphs. Pathway identifier (ID) and name are drawn from the MaizeCyc database (https://www.maizegdb.org/metabolic_pathways/).
**Table S5.** Pathways associated with decreased oil and FA concentration with enrichment scores significant at *P *<* *0.05. Pathway identifier (ID) and name are drawn from the MaizeCyc database (https://www.maizegdb.org/metabolic_pathways/), and *P* and FDR were calculated in this study.
**Table S6.** Pathways associated with effects on the ratio of different FA traits with enrichment scores significant at *P *<* *0.01. Pathway identifier (ID) and name are drawn from the MaizeCyc database (https://www.maizegdb.org/metabolic_pathways/), and *P* and FDR were calculated in this study. (https://www.maizegdb.org/metabolic_pathways/).
