# Supplementary material for: Leveraging GWAS data to identify metabolic pathways and networks involved in maize lipid biosynthesis
Source: Plant J. 2019 Mar 20;98(5):853–63. doi: 10.1111/tpj.14282 (PMC6850169; doi:10.1111/tpj.14282)
Supplement: Supplementary file 1 — Table S1. Summary of significantly associated genes from the GWAS study of Li et al. (2013), and those that were also identified by pathway analysis. Pathway identifier (ID) and name are drawn from the MaizeCyc database (https://www.maizegdb.org/metabolic_pathways/). Table S2. Summary of significantly associated SNPs from the GWAS study of Li et al. (2013) with R 2 values, and the number of tagSNPs and genes from the present study. Table S3. Pathways associated with increased FA content and with enrichment scores that were significant at 0.01 < P < 0.05. Pathway identifier (ID) and name are drawn from the MaizeCyc database (https://www.maizegdb.org/metabolic_pathways/), and P and FDR were calculated in this study. Table S4. Number of genes which contributed to the enrichment score in significant pathways at P < 0.01, and which are shown as hatch marks at the top of the running enrichment score graphs. Pathway identifier (ID) and name are drawn from the MaizeCyc database (https://www.maizegdb.org/metabolic_pathways/). Table S5. Pathways associated with decreased oil and FA concentration with enrichment scores significant at P < 0.05. Pathway identifier (ID) and name are drawn from the MaizeCyc database (https://www.maizegdb.org/metabolic_pathways/), and P and FDR were calculated in this study. Table S6. Pathways associated with effects on the ratio of different FA traits with enrichment scores significant at P < 0.01. Pathway identifier (ID) and name are drawn from the MaizeCyc database (https://www.maizegdb.org/metabolic_pathways/), and P and FDR were calculated in this study. (https://www.maizegdb.org/metabolic_pathways/). [file TPJ-98-853-s001.docx]

**Supplementary Table 1.** Summary of significantly associated genes from the GWAS study of Li et al., (2013), and those that were also identified by pathway analysis. Pathway identifier (ID) and name are drawn from the MaizeCyc database (https://www.maizegdb.org/metabolic_pathways/).

| GWAS Candidate gene, B73 V3 ID | GWAS Candidate gene, B73 V4 ID | GWAS_lead trait from Li et al., 2013^a^ | Pathway_trait from the current study | MaizeCyc ID^b^ | Effect direction^c^ |
| --- | --- | --- | --- | --- | --- |
| GRMZM2G445791 | Zm00001d029176 | C22:0/C24:0 |  | PWY-6837 |  |
| GRMZM2G062357 | Zm00001d034519 | C22:0/C24:0 | C18:0 | PWY0-1313 | ↑ |
| GRMZM2G167428 | Zm00001d034779 | C18:1/C18:2 |  | No pathway information available |  |
| GRMZM2G132468 | Zm00001d007942 | C22:0/C24:0 |  | No pathway information available |  |
| GRMZM2G044625 | Zm00014a038898 | C22:0/C24:0 |  | No pathway information available |  |
| GRMZM2G037334 | Zm00001d051367 | C18:3 |  | No pathway information available |  |
| GRMZM2G040452 | Zm00001d053566 | C16:0/C16:1 |  | No pathway information available |  |
| GRMZM2G400281 | Zm00001d013743 | C20:0/C20:1 |  | No pathway information available |  |
| GRMZM2G141999 | Zm00001d039175 | C22:0/C24:0 |  | No pathway information available |  |
| GRMZM2G153569 | Zm00001d010431 | C18:1/C18:2 |  | No pathway information available |  |
| GRMZM2G166383 | Zm00001d011756 | C22:0/C24:0 |  | No pathway information available |  |
| GRMZM2G080524 | Zm00001d027887 | Oil |  | LIPAS-PWY |  |
| GRMZM2G410515 | Zm00007a00011376 | Oil |  | No pathway information available |  |
| GRMZM2G115615 | Zm00001d031002 | Oil |  | No pathway information available |  |
| GRMZM2G110298 | Zm00001d033149 | Oil |  | No pathway information available |  |
| GRMZM2G134432 | Zm00001d003723 | Oil |  | No pathway information available |  |
| GRMZM2G079236 | Zm00001d005019 | Oil | C16:0; C16:0/C18:0; C18:0/C18:1; Oil | PWY-5143; PWY-5147; PWY-5156; PWY-5971; PWY-5995; PWY-6803 | ↑; ↓; ↓; ↑ |
| GRMZM2G176542 | Zm00001d042487 | Oil |  | No pathway information available |  |
| GRMZM2G118423 | no new ID | Oil |  | No pathway information available |  |
| GRMZM2G083195 | Zm00001d042813 | Oil | C18:2; C18:3; Oil | PHOSLIPSYN2-PWY; PWY-5667; PWY4FS-8; TRIGLSYN-PWY | ↑; ↑; ↑ |
| GRMZM2G133675 | Zm00001d048901 | Oil |  | No pathway information available |  |
| GRMZM5G847159 | Zm00001d049551 | Oil |  | No pathway information available |  |
| GRMZM2G122767 | Zm00001d051742 | Oil |  | No pathway information available |  |
| GRMZM2G125268 | Zm00001d051754 | Oil |  | No pathway information available |  |
| GRMZM2G092321 | no new ID | Oil |  | No pathway information available |  |
| GRMZM2G041060 | Zm00001d053805 | Oil |  | No pathway information available |  |
| GRMZM2G065194 | Zm00001d013651 | Oil |  | PWY-5068 |  |
| GRMZM2G439195 | Zm00001d013655 | Oil | C16:0; C16:0/C18:0; C18:1; Oil | PWY-5912; PWY-5957 | ↑; ↓; ↑; ↑ |
| GRMZM2G035779 | Zm00001d013957 | Oil |  | No pathway information available |  |
| GRMZM2G169089 | Zm00001d036982 | Oil | C18:3; Oil | TRIGLSYN-PWY | ↓; ↑ |
| GRMZM2G092550 | Zm00001d020416 | Oil |  | No pathway information available |  |
| GRMZM2G136072 | Zm00001d008883 | Oil |  | No pathway information available |  |
| GRMZM2G003022 | Zm00001d009150 | Oil |  | No pathway information available |  |
| GRMZM2G052855 | Zm00001d010174 | Oil |  | No pathway information available |  |
| GRMZM2G169240 | Zm00001d023769 | Oil | C16:0; C18:0/C18:1; Oil | PWY-5995; PWY-5995; PWY-5995 | ↑; ↓; ↑ |
| GRMZM2G162972 | no new ID | Oil |  | No pathway information available |  |
| GRMZM5G828253 | no new ID | Oil |  | No pathway information available |  |
| GRMZM2G031790 | Zm00001d027766 | C20:1 | C18:3; C18:1 | PWY-5080 | ↓; ↓ |
| GRMZM2G020523 | Zm00001d029604 | C16:1 |  | No pathway information available |  |
| GRMZM2G094742 | Zm00007a00032832 | C16:1 |  | No pathway information available |  |
| GRMZM2G059029 | Zm00001d031191 | C20:0 |  | No pathway information available |  |
| GRMZM2G152438 | Zm00001d005188 | C18:3 |  | No pathway information available |  |
| GRMZM2G169095 | Zm00001d007422 | C18:0 |  | No pathway information available |  |
| GRMZM2G142315 | Zm00001d042275 | C18:0/C18:1 |  | No pathway information available |  |
| GRMZM2G148555 | Zm00001d044191 | C16:1 |  | No pathway information available |  |
| GRMZM2G064701 | Zm00007a00009878 | C18:1 |  | No pathway information available |  |
| GRMZM2G021339 | Zm00001d051749 | C180/C181 |  | No pathway information available |  |
| GRMZM2G308193 | Zm00001d052193 | C18:0 |  | No pathway information available |  |
| GRMZM2G142342 | Zm00001d052654 | C16:0 |  | No pathway information available |  |
| GRMZM2G477205 | Zm00001d053212 | C18:0 |  | No pathway information available |  |
| GRMZM2G077295 | Zm00001d013301 | C16:0 |  | No pathway information available |  |
| GRMZM2G452189 | Zm00001d018196 | C20:0/C22:0 |  | No pathway information available |  |
| GRMZM2G105398 | Zm00001d036905 | C18:0/C20:0 |  | No pathway information available |  |
| GRMZM2G079308 | Zm00001d036919 | C20:0 | C16:0; C18:0; C18:1 | PWY-5142; PWY-5147; PWY-5156; PWY-5366; PWY-5367; PWY-5971; PWY-5973; PWY-5989 | ↑; ↑; ↑ |
| GRMZM2G032028 | Zm00001d036950 | C22:0 |  | No pathway information available |  |
| GRMZM2G101707 | Zm00007a00010573 | C24:0 |  | No pathway information available |  |
| GRMZM2G103526 | Zm00001d036978 | C20:1 | C18:0/C18:1 | PWY-5083; PWY-6357; PWY-6348 | ↑ |
| GRMZM2G029685 | Zm00001d036985 | C22:0 |  | No pathway information available |  |
| GRMZM2G416750 | Zm00001d037095 | C20:0 |  | No pathway information available |  |
| GRMZM2G138842 | Zm00001d037067 | C20:0 |  | No pathway information available |  |
| GRMZM2G701082 | Zm00001d037239 | C18:0/C18:1 |  | No pathway information available |  |
| GRMZM2G122577 | Zm00001d037515 | C18:2 |  | No pathway information available |  |
| GRMZM2G084812 | Zm00001d037540 | C18:0 |  | No pathway information available |  |
| GRMZM2G101515 | Zm00001d039041 | C20:1 |  | No pathway information available |  |
| GRMZM2G350265 | Zm00007a00034043 | C18:1 |  | No pathway information available |  |
| AC204277.3_FG006 | Zm00001d010599 | C20:0 |  | PWY-6745 |  |
| GRMZM2G025966 | Zm00001d011758 | C18:0 |  | No pathway information available |  |
| GRMZM5G863550 | Zm00001d045324 | C16:0 | C18:2 | PHOSLIPSYN2-PWY; PWY-5269; PWY4FS-7; PWY4FS-8 | ↑ |
| GRMZM2G005522 | Zm00001d045338 | SFA/USFA |  | PWY-5859 |  |
| GRMZM5G829544 | Zm00001d045387 | C16:0 | C16:0; C18:0; C18:1 | PWY-5142; PWY-5147; PWY-5156; PWY-5366; PWY-5367; PWY-5971; PWY-5973; PWY-5989 | ↑; ↑; ↑ |
| GRMZM2G022558 | Zm00001d046444 | C18:0/C20:0 | C18:3; C18:1 | PWY-5080 | ↓; ↓ |
| GRMZM2G117207 | Zm00001d047745 | C18:2/C18:3 |  | No pathway information available |  |
| GRMZM2G434170 | Zm00007a00028206 | C18:1/C18:2 |  | No pathway information available |  |
| GRMZM2G044306 | Zm00001d024583 | C18:0 |  | No pathway information available |  |
| ^a^The oil trait associated with the highest P value among oil compositional traits from the GWAS study of Li et al., (2013) | | | | |  |
| ^b^ The significant pathways (at P<0.01) in which GWAS identified candidate genes contribute to the enrichment score, from the V3 and V4 B73 reference sequences. | | | | | |
| ^c^The pathway effect direction on corresponding oil-related trait.↑, increasing corresponding oil-related trait; ↓,decreasing corresponding oil-related trait. | | | | | |

**Supplementary Table 2.** Summary of significantly associated SNPs from the GWAS study of Li et al., (2013) with *R^2^* values, and the number of tagSNPs and genes from the present study.

| Trait^a^ | # of  SNPs^b^ | Largest  *R*^2^ | Total # of tagSNPs | # of mapped tagSNPs^c^ | # of mapped genes^d^ |
| --- | --- | --- | --- | --- | --- |
| Oil | 139 | 0.18 | 22365 | 192 | 328 |
| C16:0 | 64 | 0.16 | 26286 | 200 | 251 |
| C18:0 | 98 | 0.22 | 26336 | 198 | 247 |
| C18:1 | 95 | 0.24 | 26304 | 252 | 299 |
| C18:2 | 78 | 0.14 | 26289 | 140 | 168 |
| C18:3 | 4 | 0.08 | 26344 | 321 | 406 |
| C16:0/C18:0 | 20 | 0.10 | 26324 | 215 | 254 |
| C18:0/C18:1 | 8 | 0.08 | 26324 | 340 | 458 |
| C18:1/C18:2 | 48 | 0.20 | 26297 | 259 | 320 |
| C18:2/C18:3 | 0 | 0.07 | 26337 | 301 | 375 |

^a^Ten oil-related traits were run in this pathway analysis. They are oil content, the FA concentration of palmitic (C16:0), stearic (C18:0), oleic (C18:1), linoleic (C18:2), and the ratios of palmitic to stearic (C16:0/C18:0), stearic to oleic (C18:0/C18:1), oleic to linoleic (C18:1/C18:2), and linoleic to linolenic (C18:2/C18:3). ^b^The number of significantly associated SNPs for oil-related traits at *P* < 1.8 × 10^-6^ from previous GWAS results (Li et al., 2013). ^c^The number of tagSNPs mapped to significant pathways with ≥ 5 genes in the study of Li et al. (2013). ^d^The number of genes mapped to significant pathways with ≥ 5 genes in the current study.

**Supplementary Table 3.** Pathways associated with increased FA content and with enrichment scores that were significant at 0.01 < *P* < 0.05. Pathway identifier (ID) and name are drawn from the MaizeCyc database (<https://www.maizegdb.org/metabolic_pathways/>), and *P* and FDR were calculated in this study.

| MaizeCyc ID | Lead trait^a^ | Other trait^b^ | Pathway name | Lead trait *P* value | Lead trait FDR |
| --- | --- | --- | --- | --- | --- |
| ALACAT2-PWY | C18:3 |  | alanine degradation II (to D-lactate) | 0.01691 | 0.49559 |
| ALANINE-SYN2-PWY | C18:3 |  | alanine biosynthesis II | 0.01742 | 0.49559 |
| CITRULBIO-PWY | C18:3 |  | citrulline biosynthesis | 0.01474 | 0.49559 |
| COA-PWY-1 | C18:2 |  | coenzyme A biosynthesis | 0.04646 | 0.61523 |
| ETHYL-PWY | C18:0 |  | ethylene biosynthesis from methionine | 0.04757 | 0.60759 |
| FASYN-ELONG-PWY | C16:0 |  | fatty acid elongation -- saturated | 0.03024 | 0.44790 |
| GLYOXYLATE-BYPASS | C18:0 |  | glyoxylate cycle | 0.01511 | 0.39401 |
| LEU-DEG2-PWY | C16:0 | C18:1,C18:3 | leucine degradation I | 0.01797 | 0.35148 |
| LEUSYN-PWY | C18:1 | C16:0 | leucine biosynthesis | 0.03790 | 0.50532 |
| MALATE-ASPARTATE-SHUTTLE-PWY | C18:1 |  | aspartate degradation II | 0.04139 | 0.51821 |
| PHOSLIPSYN2-PWY | C18:2 |  | phospholipid biosynthesis II | 0.02996 | 0.61523 |
| PROSYN-PWY | C18:3 |  | proline biosynthesis I | 0.01515 | 0.49559 |
| PWY0-1313 | C18:0 |  | acetate conversion to acetyl-CoA | 0.04598 | 0.60759 |
| PWY0-163 | C18:1 | C18:0,C18:2 | salvage pathways of pyrimidine ribonucleotides | 0.01443 | 0.50002 |
| PWY0-166 | C18:2 |  | pyrimidine deoxyribonucleotides *de novo* biosynthesis I | 0.03024 | 0.61523 |
| PWY-1001 | C18:2 |  | cellulose biosynthesis | 0.02785 | 0.61523 |
| PWY-102 | C18:0 |  | gibberellin inactivation I (2*β*;-hydroxylation) | 0.03533 | 0.56020 |
| PWY-1042 | C16:0 |  | glycolysis IV (plant cytosol) | 0.04841 | 0.56123 |
| PWY1F-353 | C18:2 |  | glycine betaine biosynthesis III (plants) | 0.02250 | 0.61523 |
| PWY1F-467 | C18:1 |  | phenylpropanoid biosynthesis, initial reactions | 0.01689 | 0.50002 |
| PWY1F-FLAVSYN | C18:2 |  | flavonoid biosynthesis | 0.03358 | 0.61523 |
| PWY-3101 | C18:3 |  | flavonol biosynthesis | 0.02964 | 0.55629 |
| PWY-3461 | C18:2 |  | tyrosine biosynthesis II | 0.04219 | 0.61523 |
| PWY-381 | C18:2 |  | nitrate reduction II (assimilatory) | 0.02889 | 0.61523 |
| PWY-4081 | C16:0 |  | glutathione redox reactions I | 0.02857 | 0.44790 |
| PWY-4221-1 | C16:0 |  | superpathway of pantothenate and coenzyme A biosynthesis II | 0.01582 | 0.34125 |
| PWY4FS-5 | C18:3 |  | superpathway of phosphatidylcholine biosynthesis | 0.04228 | 0.55629 |
| PWY-5004 | C18:3 |  | superpathway of citrulline metabolism | 0.03582 | 0.55629 |
| PWY-5035 | C18:3 |  | gibberellin biosynthesis III (early C-13 hydroxylation) | 0.03139 | 0.55629 |
| PWY-5041 | C18:0 | C16:0,C18:2,C18:3 | *S*-adenosyl-L-methionine cycle II | 0.01279 | 0.39401 |
| PWY-5053 | C18:1 |  | superpathway of GA_12_ biosynthesis | 0.03875 | 0.50532 |
| PWY-5057 | C18:0 |  | valine degradation II | 0.03580 | 0.56020 |
| PWY-5059 | C18:2 |  | pinobanksin biosynthesis | 0.04456 | 0.61523 |
| PWY-5060 | C18:3 |  | luteolin biosynthesis | 0.02216 | 0.55629 |
| PWY-5076 | C18:0 |  | leucine degradation III | 0.03580 | 0.56020 |
| PWY-5098 | C16:0 |  | chlorophyll *a* degradation | 0.04138 | 0.53578 |
| PWY-5121 | C18:1 |  | superpathway of geranylgeranyldiphosphate biosynthesis II (via MEP) | 0.02603 | 0.50002 |
| PWY-5122 | C18:3 |  | geranyl diphosphate biosynthesis | 0.02810 | 0.55629 |
| PWY-5129 | C18:2 |  | sphingolipid biosynthesis (plants) | 0.02099 | 0.61523 |
| PWY-5142 | C16:0 | C18:1 | acyl-ACP thioesterase pathway | 0.01444 | 0.34125 |
| PWY-5147 | C18:0 | C18:2 | oleate biosynthesis I (plants) | 0.02342 | 0.48876 |
| PWY-5168 | C18:0 |  | ferulate and sinapate biosynthesis | 0.03544 | 0.56020 |
| PWY-5173 | C18:0 | C18:2 | superpathway of acetyl-CoA biosynthesis | 0.03309 | 0.56020 |
| PWY-5269 | C18:2 |  | cardiolipin biosynthesis II | 0.04717 | 0.61523 |
| PWY-5278 | C18:1 |  | sulfite oxidation III | 0.03043 | 0.50002 |
| PWY-5340 | C18:3 | C16:0,C18:1 | sulfate activation for sulfonation | 0.02590 | 0.55629 |
| PWY-5366 | C16:0 | C18:1 | palmitoleate biosynthesis II | 0.01444 | 0.34125 |
| PWY-5461 | C18:1 |  | betanidin degradation | 0.02804 | 0.50002 |
| PWY-5464 | C16:0 | C18:0 | superpathway of cytosolic glycolysis (plants), pyruvate dehydrogenase and TCA cycle | 0.01635 | 0.34125 |
| PWY-5800 | C18:2 |  | xylan biosynthesis | 0.02103 | 0.61523 |
| PWY-5885 | C18:1 |  | wax esters biosynthesis II | 0.01746 | 0.50002 |
| PWY-5912 | C18:0 | C18:2,C18:3 | 2'-deoxymugineic acid phytosiderophore biosynthesis | 0.01435 | 0.39401 |
| PWY-5934 | C18:2 |  | Fe(III)-reduction and Fe(II) transport | 0.04130 | 0.61523 |
| PWY-5995 | C18:2 | C18:1 | linoleate biosynthesis I (plants) | 0.03030 | 0.61523 |
| PWY-6118 | C18:3 |  | glycerol-3-phosphate shuttle | 0.04706 | 0.56648 |
| PWY-6121 | C18:1 | C18:3 | 5-aminoimidazole ribonucleotide biosynthesis I | 0.02721 | 0.50002 |
| PWY-6122 | C18:3 | C18:1 | 5-aminoimidazole ribonucleotide biosynthesis II | 0.01373 | 0.49559 |
| PWY-6151 | C18:1 |  | *S*-adenosyl-L-methionine cycle I | 0.04848 | 0.53752 |
| PWY-6164 | C18:3 |  | 3-dehydroquinate biosynthesis I | 0.03351 | 0.55629 |
| PWY-6277 | C18:1 | C18:3 | superpathway of 5-aminoimidazole ribonucleotide biosynthesis | 0.02721 | 0.50002 |
| PWY-6352 | C18:3 |  | 3-phosphoinositide biosynthesis | 0.03533 | 0.55629 |
| PWY-6475-1 | C16:0 |  | *trans*-lycopene biosynthesis II (plants) | 0.03047 | 0.44790 |
| PWY66-21 | C18:0 | C16:0 | ethanol degradation II (cytosol) | 0.01777 | 0.42780 |
| PWY-6629 | C18:3 |  | superpathway of tryptophan biosynthesis | 0.04443 | 0.55629 |
| SAM-PWY | C18:1 |  | S-adenosyl-L-methionine biosynthesis | 0.04456 | 0.52598 |
| SO4ASSIM-PWY | C18:3 | C16:0,C18:1 | sulfate reduction I (assimilatory) | 0.02590 | 0.55629 |
| SULFMETII-PWY | C16:0 | C18:1,C18:2 | sulfate reduction II (assimilatory) | 0.01189 | 0.33840 |

^a^The FA trait with the most significant *P* value. ^b^Additional FA trait with significant *P* value (0.01 < *P* < 0.05).

**Supplementary Table 4.** Number of genes which contributed to the enrichment score in significant pathways at *P* < 0.01, and which are shown as hatch marks at the top of the running enrichment score graphs. Pathway identifier (ID) and name are drawn from the MaizeCyc database (https://www.maizegdb.org/metabolic_pathways/).

| MaizeCyc ID | Trait | Effect direction^a^ | # of contributing genes^b^ |
| --- | --- | --- | --- |
| PWY-6605 | Oil | ↓ | 10 |
| PWY-3841 | Oil | ↓ | 17 |
| PWY-4821 | Oil | ↓ | 18 |
| GLYSYN-PWY | Oil | ↓ | 8 |
| PWY-6196 | Oil | ↓ | 11 |
| SER-GLYSYN-PWY | Oil | ↓ | 13 |
| PWY-2161 | Oil | ↓ | 17 |
| PWY-1081 | Oil | ↓ | 62 |
| BSUBPOLYAMSYN-PWY | Oil | ↓ | 9 |
| PWY-5121 | Oil | ↑ | 39 |
| PWY-5995 | Oil | ↑ | 16 |
| PWY-5143 | Oil | ↑ | 13 |
| ARGSPECAT-PWY | C160 | ↓ | 7 |
| PWY-4821 | C160 | ↓ | 20 |
| PWY-3841 | C160 | ↓ | 17 |
| GLYSYN-PWY | C160 | ↓ | 8 |
| SER-GLYSYN-PWY | C160 | ↓ | 14 |
| PWY-6196 | C160 | ↓ | 11 |
| PWY-2161 | C160 | ↓ | 17 |
| BSUBPOLYAMSYN-PWY | C160 | ↓ | 9 |
| PWY-801 | C160 | ↓ | 12 |
| PWY-5941 | C160 | ↓ | 16 |
| GLYCOCAT-PWY | C160 | ↓ | 26 |
| PWY-5995 | C160 | ↑ | 18 |
| PWY-5971 | C160 | ↑ | 40 |
| PWY-5973 | C160 | ↑ | 39 |
| PWY-5912 | C160 | ↑ | 10 |
| PWY-5367 | C160 | ↑ | 39 |
| PWY-5156 | C160 | ↑ | 53 |
| PWY-5989 | C160 | ↑ | 40 |
| PWY-5147 | C160 | ↑ | 15 |
| COA-PWY-1 | C160 | ↑ | 8 |
| PWY-5035 | C160 | ↑ | 7 |
| PWY-801 | C180 | ↓ | 12 |
| PWY-2841 | C180 | ↓ | 10 |
| PWY-4261 | C180 | ↓ | 12 |
| GLYCOCAT-PWY | C180 | ↓ | 26 |
| PWY-6118 | C180 | ↓ | 20 |
| PWY-842 | C180 | ↓ | 57 |
| PWY-6596 | C180 | ↓ | 27 |
| PWY-6606 | C180 | ↓ | 12 |
| PWY-5971 | C180 | ↑ | 41 |
| PWY-5973 | C180 | ↑ | 39 |
| PWY-5367 | C180 | ↑ | 39 |
| PWY-6151 | C180 | ↑ | 11 |
| PWY-5142 | C180 | ↑ | 7 |
| FASYN-ELONG-PWY | C180 | ↑ | 34 |
| PWY-5156 | C180 | ↑ | 54 |
| PWY-5366 | C180 | ↑ | 7 |
| PWY-5989 | C180 | ↑ | 41 |
| PWY-401 | C181 | ↓ | 8 |
| PWY-5918 | C181 | ↓ | 48 |
| PWY-801 | C181 | ↓ | 12 |
| PWY-6606 | C181 | ↓ | 12 |
| PWY-6118 | C181 | ↓ | 20 |
| PWY-2161 | C181 | ↓ | 17 |
| MANNOSYL-CHITO-DOLICHOL-BIOSYNTHESIS | C181 | ↓ | 7 |
| PWY-5147 | C181 | ↑ | 15 |
| PWY-6628 | C181 | ↑ | 70 |
| PWY-4081 | C181 | ↑ | 7 |
| PWY-6629 | C181 | ↑ | 76 |
| PWY-6457 | C181 | ↑ | 8 |
| ARO-PWY | C181 | ↑ | 49 |
| PWY-5912 | C181 | ↑ | 10 |
| PWY-2161 | C182 | ↓ | 17 |
| PWY-2781 | C182 | ↓ | 8 |
| ILEUSYN-PWY | C182 | ↓ | 33 |
| PWY-4821 | C182 | ↓ | 20 |
| PWY-3841 | C182 | ↓ | 17 |
| ARGDEG-V-PWY | C182 | ↓ | 10 |
| BSUBPOLYAMSYN-PWY | C182 | ↓ | 9 |
| SO4ASSIM-PWY | C182 | ↑ | 8 |
| PWY-5687 | C182 | ↑ | 18 |
| PWY-5340 | C182 | ↑ | 8 |
| UDPNACETYLGALSYN-PWY | C182 | ↑ | 12 |
| PWY0-1325 | C183 | ↓ | 5 |
| PWY-2161 | C183 | ↓ | 17 |
| SER-GLYSYN-PWY | C183 | ↓ | 14 |
| PWY-3841 | C183 | ↓ | 17 |
| GLYSYN-PWY | C183 | ↓ | 8 |
| PWY-801 | C183 | ↓ | 12 |
| PWY-5464 | C183 | ↓ | 208 |
| ASPARAGINE-BIOSYNTHESIS | C183 | ↓ | 5 |
| GLUCONEO-PWY | C183 | ↓ | 101 |
| PWY-5080 | C183 | ↓ | 13 |
| PWY-5934 | C183 | ↑ | 6 |
| PWY-381 | C183 | ↑ | 13 |
| PYRIDNUCSYN-PWY-1 | C183 | ↑ | 6 |
| PWY-3282 | C183 | ↑ | 12 |
| PWY1F-353 | C183 | ↑ | 5 |
| PWY-5912 | C160/C180 | ↓ | 10 |
| PWY-6151 | C160/C180 | ↓ | 11 |
| PWY-6457 | C160/C180 | ↓ | 8 |
| PWY-5143 | C160/C180 | ↓ | 13 |
| RIBOSYN2-PWY-1 | C160/C180 | ↓ | 13 |
| PWY-6549 | C160/C180 | ↓ | 35 |
| PWY-6352 | C160/C180 | ↓ | 14 |
| PWY-5168 | C160/C180 | ↓ | 7 |
| PWY-5123 | C160/C180 | ↓ | 25 |
| PWY-5122 | C160/C180 | ↓ | 20 |
| PWY-5098 | C160/C180 | ↑ | 6 |
| NONOXIPENT-PWY | C160/C180 | ↑ | 15 |
| PWY-5381-1 | C160/C180 | ↑ | 12 |
| PWY-6440 | C160/C180 | ↑ | 8 |
| P21-PWY | C160/C180 | ↑ | 5 |
| PWY-5059 | C160/C180 | ↑ | 24 |
| PENTOSE-P-PWY | C160/C180 | ↑ | 35 |
| PWY-5995 | C180/C181 | ↓ | 18 |
| PWY-6121 | C180/C181 | ↓ | 13 |
| ARO-PWY | C180/C181 | ↓ | 49 |
| PWY-6277 | C180/C181 | ↓ | 13 |
| PWY-5278 | C180/C181 | ↓ | 8 |
| PWY-6357 | C180/C181 | ↑ | 34 |
| PWY-6299 | C180/C181 | ↑ | 8 |
| PWY-6126 | C180/C181 | ↑ | 112 |
| TRESYN-PWY | C180/C181 | ↑ | 23 |
| PWY-6348 | C180/C181 | ↑ | 34 |
| PWY-5083 | C180/C181 | ↑ | 94 |
| PWY-6352 | C180/C181 | ↑ | 14 |
| SALVADEHYPOX-PWY | C180/C181 | ↑ | 10 |
| PWY0-162 | C181/C182 | ↓ | 33 |
| CYSTSYN-PWY | C181/C182 | ↓ | 16 |
| PWY-5129 | C181/C182 | ↓ | 11 |
| PWY-801 | C181/C182 | ↓ | 12 |
| PWY-3101 | C181/C182 | ↓ | 16 |
| PWY-6605 | C181/C182 | ↓ | 10 |
| PWY-3561 | C181/C182 | ↓ | 18 |
| PWY-5060 | C181/C182 | ↓ | 11 |
| SER-GLYSYN-PWY | C181/C182 | ↓ | 14 |
| PWY-6629 | C181/C182 | ↑ | 76 |
| ARO-PWY | C181/C182 | ↑ | 49 |
| PWY-5121 | C181/C182 | ↑ | 47 |
| PWY-5123 | C181/C182 | ↑ | 25 |
| PWY-6628 | C181/C182 | ↑ | 70 |
| FOLSYN-PWY-1 | C182/C183 | ↓ | 16 |
| BSUBPOLYAMSYN-PWY | C182/C183 | ↓ | 9 |
| TRNA-CHARGING-PWY | C182/C183 | ↓ | 69 |
| PWY0-163 | C182/C183 | ↑ | 43 |
| PWY-1042 | C182/C183 | ↑ | 140 |
| PWY-6619 | C182/C183 | ↑ | 9 |
| PWY-5687 | C182/C183 | ↑ | 18 |
| GLYCOLYSIS | C182/C183 | ↑ | 148 |

^a^The pathway effect direction on corresponding oil-related trait.↑, increasing corresponding oil-related trait; ↓,decreasing corresponding oil-related trait. ^b^The number of hatch marks genes which contributed to the enrichment score in significant pathways at P < 0.01

**Supplementary Table 5.** Pathways associated with decreased oil and FA concentration with enrichment scores significant at *P* < 0.05. Pathway identifier (ID) and name are drawn from the MaizeCyc database (<https://www.maizegdb.org/metabolic_pathways/>), and *P* and FDR were calculated in this study.

| MaizeCyc ID | Lead trait^a^ | Other trait^b^ | Pathway name | Lead trait *P* value | Lead trait FDR |
| --- | --- | --- | --- | --- | --- |
| ARGSYNBSUB-PWY | Oil |  | arginine biosynthesis II (acetyl cycle) | 0.04865 | 0.66340 |
| ARGSYN-PWY | Oil |  | arginine biosynthesis I | 0.04192 | 0.62877 |
| GLYSYN-PWY | Oil | C18:3, C16:0, C18:1, C18:2 | glycine biosynthesis I | 0.00046 | 0.05322 |
| LIPASYN-PWY | Oil | C18:1 | phospholipases | 0.03721 | 0.62022 |
| PWY-1081 | Oil |  | homogalacturonan degradation | 0.00819 | 0.29839 |
| PWY-2161 | Oil | C16:0, C18:1, C18:2, C18:3 | folate polyglutamylation | 0.00022 | 0.05322 |
| PWY-4821 | Oil | C16:0, C18:0,C18:2, C18:3 | UDP-D-xylose and UDP-D-glucuronate biosynthesis | 0.00053 | 0.05322 |
| PWY-5686 | Oil |  | uridine-5'-phosphate biosynthesis | 0.04028 | 0.62877 |
| PWY-6196 | Oil | C16:0, C18:0, C18:2 | serine racemization | 0.00722 | 0.29839 |
| PWY-6605 | Oil | C16:0 | adenine and adenosine salvage II | 0.00895 | 0.29839 |
| SER-GLYSYN-PWY | Oil | C16:0, C18:3 | superpathway of serine and glycine biosynthesis I | 0.00087 | 0.06503 |
| TRESYN-PWY | Oil |  | trehalose biosynthesis I | 0.03484 | 0.61488 |
| ARGSPECAT-PWY | C16:0 | Oil, C18:2 | spermine biosynthesis | 0.00502 | 0.25873 |
| ASPARTATESYN-PWY | C16:0 |  | aspartate biosynthesis | 0.02640 | 0.37566 |
| PWY-282 | C16:0 | C18:0 | cuticular wax biosynthesis | 0.01993 | 0.37566 |
| PWY-3001 | C16:0 | C18:2 | isoleucine biosynthesis I | 0.02315 | 0.37566 |
| PWY-5941 | C16:0 | C18:0 | glycogen degradation II | 0.00588 | 0.25873 |
| PWY-6126 | C16:0 |  | adenosine nucleotides *de novo* biosynthesis | 0.02513 | 0.37566 |
| PWY-621 | C16:0 |  | sucrose degradation III | 0.03339 | 0.41802 |
| PWY-6556 | C16:0 | Oil | pyrimidine ribonucleosides degradation II | 0.02158 | 0.37566 |
| PWY-6622 | C16:0 | C18:0 | heptadecane biosynthesis | 0.01286 | 0.26839 |
| SERSYN-PWY | C16:0 |  | serine biosynthesis | 0.01204 | 0.26839 |
| THIOREDOX-PWY | C16:0 | C18:0 | thioredoxin pathway | 0.04350 | 0.47729 |
| GLUCOSE1PMETAB-PWY | C18:0 | C16:0, C18:3 | glucose and glucose-1-phosphate degradation | 0.01594 | 0.34334 |
| GLYCOCAT-PWY | C18:0 | C16:0 | glycogen degradation I | 0.00881 | 0.34334 |
| PWY1F-823 | C18:0 |  | leucopelargonidin and leucocyanidin biosynthesis | 0.01755 | 0.34334 |
| PWY-2841 | C18:0 |  | cytokinins degradation | 0.00878 | 0.34334 |
| PWY-4261 | C18:0 | C18:1 | glycerol degradation IV | 0.00038 | 0.09722 |
| PWY-5053 | C18:0 |  | superpathway of GA_12_ biosynthesis | 0.04903 | 0.55080 |
| PWY-5152 | C18:0 |  | leucodelphinidin biosynthesis | 0.01755 | 0.34334 |
| PWY-5661 | C18:0 | C16:0,C18:3 | GDP-glucose biosynthesis | 0.01594 | 0.34334 |
| PWY-5767 | C18:0 |  | glycogen degradation III | 0.01402 | 0.34334 |
| PWY-6118 | C18:0 | C18:1 | glycerol-3-phosphate shuttle | 0.00387 | 0.30311 |
| PWY-6440 | C18:0 | Oil, C18:1 | spermine and spermidine degradation II | 0.01146 | 0.34334 |
| PWY-6596 | C18:0 | C18:1 | adenosine nucleotides degradation I | 0.00062 | 0.09722 |
| PWY-842 | C18:0 | C16:0 | starch degradation | 0.00104 | 0.10843 |
| XYLCAT-PWY | C18:0 |  | xylose degradation I | 0.02781 | 0.48364 |
| BGALACT-PWY | C18:1 |  | lactose degradation III | 0.02621 | 0.51279 |
| CYSTSYN-PWY | C18:1 |  | cysteine biosynthesis I | 0.01340 | 0.38328 |
| HEME-BIOSYNTHESIS-II | C18:1 |  | heme biosynthesis from uroporphyrinogen-III I | 0.01398 | 0.38328 |
| MANNOSYL-CHITO-DOLICHOL-BIOSYNTHESIS | C18:1 | Oil, C18:0, C18:2 | dolichyl-diphosphooligosaccharide biosynthesis | 0.00796 | 0.38328 |
| PWY-181 | C18:1 | Oil | photorespiration | 0.02136 | 0.47750 |
| PWY-2681 | C18:1 | C16:0 | *trans*-zeatin biosynthesis | 0.03786 | 0.62896 |
| PWY-401 | C18:1 | C18:2 | glycolipid biosynthesis | 0.00939 | 0.38328 |
| PWY-5918 | C18:1 |  | heme biosynthesis I | 0.00935 | 0.38328 |
| PWY-6606 | C18:1 | Oil, C16:0, C18:0, C18:3 | guanosine nucleotides degradation II | 0.00128 | 0.22250 |
| ARGDEG-V-PWY | C18:2 |  | arginine degradation X (arginine monooxygenase pathway) | 0.00387 | 0.24243 |
| BRANCHED-CHAIN-AA-SYN-PWY | C18:2 |  | superpathway of leucine, valine, and isoleucine biosynthesis | 0.03893 | 0.53038 |
| BSUBPOLYAMSYN-PWY | C18:2 | Oil, C16:0, C18:3 | spermidine biosynthesis I | 0.00135 | 0.17595 |
| FOLSYN-PWY-1 | C18:2 |  | superpathway of tetrahydrofolate biosynthesis | 0.04562 | 0.54000 |
| HOMOSER-THRESYN-PWY | C18:2 |  | threonine biosynthesis from homoserine | 0.04271 | 0.54000 |
| ILEUSYN-PWY | C18:2 | Oil, C16:0 | isoleucine biosynthesis I (from threonine) | 0.00553 | 0.24706 |
| PWY-2781 | C18:2 | C16:0, C18:0, C18:1 | *cis*-zeatin biosynthesis | 0.00480 | 0.24706 |
| PWY-3841 | C18:2 | Oil, C16:0, C18:3 | folate transformations II (plants) | 0.00135 | 0.17595 |
| PWY-5057 | C18:2 |  | valine degradation II | 0.01855 | 0.38717 |
| PWY-5076 | C18:2 |  | leucine degradation III | 0.01855 | 0.38717 |
| PWY-5481 | C18:2 | C18:3 | pyruvate fermentation to lactate | 0.01654 | 0.38717 |
| PWY-5486 | C18:2 |  | pyruvate fermentation to ethanol II | 0.02405 | 0.41405 |
| PWY-6333 | C18:2 |  | acetaldehyde biosynthesis I | 0.02405 | 0.41405 |
| PWY-66 | C18:2 |  | GDP-L-fucose biosynthesis I (from GDP-D-mannose) | 0.04327 | 0.54000 |
| PWY66-162 | C18:2 |  | ethanol degradation IV (peroxisomal) | 0.03233 | 0.50599 |
| PWY-6628 | C18:2 |  | superpathway of phenylalanine biosynthesis | 0.02513 | 0.41405 |
| VALSYN-PWY | C18:2 |  | valine biosynthesis | 0.03897 | 0.53038 |
| ANAGLYCOLYSIS-PWY | C18:3 |  | glycolysis III | 0.01689 | 0.34069 |
| ASPARAGINE-BIOSYNTHESIS | C18:3 |  | asparagine biosynthesis I | 0.00513 | 0.17829 |
| DETOX1-PWY | C18:3 | C16:0 | superoxide radicals degradation | 0.03338 | 0.36757 |
| GLUCONEO-PWY | C18:3 | C18:2 | gluconeogenesis I | 0.00493 | 0.17829 |
| GLYCOLYSIS | C18:3 |  | glycolysis I | 0.02359 | 0.34069 |
| GLYOXYLATE-BYPASS | C18:3 |  | glyoxylate cycle | 0.01767 | 0.34069 |
| MALATE-ASPARTATE-SHUTTLE-PWY | C18:3 |  | aspartate degradation II | 0.01568 | 0.34069 |
| PWY0-1325 | C18:3 |  | superpathway of asparagine biosynthesis | 0.00513 | 0.17829 |
| PWY-1042 | C18:3 |  | glycolysis IV (plant cytosol) | 0.02268 | 0.34069 |
| PWY-2261 | C18:3 |  | ascorbate glutathione cycle | 0.02774 | 0.36183 |
| PWY-3861 | C18:3 |  | mannitol degradation II | 0.03523 | 0.36757 |
| PWY-4361 | C18:3 |  | methionine salvage I | 0.01948 | 0.34069 |
| PWY-4541 | C18:3 | C18:0 | lipid-dependent phytate biosynthesis I (via Ins(1,4,5)P_3_) | 0.02380 | 0.34069 |
| PWY-5080 | C18:3 | C18:1 | very long chain fatty acid biosynthesis | 0.00509 | 0.17829 |
| PWY-5441 | C18:3 |  | S-methylmethionine cycle | 0.03231 | 0.36757 |
| PWY-5464 | C18:3 |  | superpathway of cytosolic glycolysis (plants), pyruvate dehydrogenase and TCA cycle | 0.00299 | 0.17829 |
| PWY-561 | C18:3 |  | superpathway of glyoxylate cycle | 0.02585 | 0.35182 |
| PWY-5690 | C18:3 |  | TCA cycle variation III (eukaryotic) | 0.02176 | 0.34069 |
| PWY-6124 | C18:3 |  | inosine-5'-phosphate biosynthesis II | 0.04665 | 0.44244 |
| PWY-622 | C18:3 |  | starch biosynthesis | 0.02395 | 0.34069 |
| PWY-6361 | C18:3 | C18:0 | 1D-*myo*-inositol hexakisphosphate biosynthesis I (from Ins(1,4,5)P3) | 0.02380 | 0.34069 |
| PWY-801 | C18:3 | Oil, C16:0, C18:0, C18:1, C18:2 | homocysteine and cysteine interconversion | 0.00074 | 0.17829 |
| TRIGLSYN-PWY | C18:3 |  | triacylglycerol biosynthesis | 0.03365 | 0.36757 |

^a^The FA trait with the most significant *P* value. ^b^Additional FA trait with significant *P* value (*P* < 0.05).

**Supplementary Table 6.** Pathways associated with effects on the ratio of different FA traits with enrichment scores significant at *P* < 0.01. Pathway identifier (ID) and name are drawn from the MaizeCyc database (<https://www.maizegdb.org/metabolic_pathways/>), and *P* and FDR were calculated in this study.

| MaizeCyc ID | Lead trait^a^ | Other trait^b^ | Effect direction^c^ | Pathway name | Lead trait *P* value | Lead trait FDR |
| --- | --- | --- | --- | --- | --- | --- |
| NONOXIPENT-PWY | C16:0/C18:0 | | ↑ | pentose phosphate pathway (non-oxidative branch) | 0.00126 | 0.24218 |
| P21-PWY | C16:0/C18:0 | | ↑ | pentose phosphate pathway (partial) | 0.00464 | 0.24218 |
| PENTOSE-P-PWY | C16:0/C18:0 | | ↑ | pentose phosphate pathway | 0.00404 | 0.24218 |
| PWY-5059 | C16:0/C18:0 | | ↑ | pinobanksin biosynthesis | 0.00424 | 0.24218 |
| PWY-5098 | C16:0/C18:0 | | ↑ | chlorophyll *α*degradation | 0.00447 | 0.24218 |
| PWY-5381-1 | C16:0/C18:0 | | ↑ | pyridine nucleotide cycling (cytosolic) | 0.00614 | 0.27436 |
| PWY-6440 | C16:0/C18:0 | | ↑ | spermine and spermidine degradation II | 0.00273 | 0.24218 |
| PWY-5122 | C16:0/C18:0 | | ↓ | geranyl diphosphate biosynthesis | 0.00406 | 0.23367 |
| PWY-5123 | C16:0/C18:0 | C18:1/C18:2 | ↓ | *trans*, *trans*-farnesyl diphosphate biosynthesis | 0.00349 | 0.23367 |
| PWY-5143 | C16:0/C18:0 | | ↓ | fatty acid activation | 0.00304 | 0.23367 |
| PWY-5168 | C16:0/C18:0 | | ↓ | ferulate and sinapate biosynthesis | 0.00718 | 0.23367 |
| PWY-5912 | C16:0/C18:0 | | ↓ | 2'-deoxymugineic acid phytosiderophore biosynthesis | 0.00684 | 0.23367 |
| PWY-6151 | C16:0/C18:0 | | ↓ | *S*-adenosyl-L-methionine cycle I | 0.00658 | 0.23367 |
| PWY-6352 | C16:0/C18:0 | C18:0/C18:1 | ↓ | 3-phosphoinositide biosynthesis | 0.00331 | 0.23367 |
| PWY-6457 | C16:0/C18:0 | | ↓ | *trans*-cinnamoyl-CoA biosynthesis | 0.00747 | 0.23367 |
| PWY-6549 | C16:0/C18:0 | | ↓ | glutamine biosynthesis III | 0.00405 | 0.23367 |
| RIBOSYN2-PWY-1 | C16:0/C18:0 | | ↓ | riboflavin biosynthesis I (plastidic) | 0.00668 | 0.23367 |
| PWY-5083 | C18:0/C18:1 | | ↑ | NAD/NADH phosphorylation and dephosphorylation | 0.00374 | 0.20397 |
| PWY-6126 | C18:0/C18:1 | | ↑ | adenosine nucleotides *de novo* biosynthesis | 0.00871 | 0.34070 |
| PWY-6299 | C18:0/C18:1 | | ↑ | aldehyde oxidation I | 0.00534 | 0.23856 |
| PWY-6348 | C18:0/C18:1 | | ↑ | phosphate acquisition | 0.00265 | 0.20397 |
| PWY-6357 | C18:0/C18:1 | | ↑ | phosphate utilization in cell wall regeneration | 0.00265 | 0.20397 |
| SALVADEHYPOX-PWY | C18:0/C18:1 | | ↑ | adenosine nucleotides degradation II | 0.00229 | 0.20397 |
| TRESYN-PWY | C18:0/C18:1 | | ↑ | trehalose biosynthesis I | 0.00043 | 0.13324 |
| PWY-5278 | C18:0/C18:1 | | ↓ | sulfite oxidation III | 0.00890 | 0.51394 |
| PWY-5995 | C18:0/C18:1 | | ↓ | linoleate biosynthesis I (plants) | 0.00916 | 0.51394 |
| PWY-6121 | C18:0/C18:1 | | ↓ | 5-aminoimidazole ribonucleotide biosynthesis I | 0.00388 | 0.51394 |
| PWY-6277 | C18:0/C18:1 | | ↓ | superpathway of 5-aminoimidazole ribonucleotide biosynthesis | 0.00388 | 0.51394 |
| ARO-PWY | C18:1/C18:2 | C18:0/C18:1 | ↑ | chorismate biosynthesis I | 0.00178 | 0.38197 |
| PWY-5121 | C18:1/C18:2 | | ↑ | superpathway of geranylgeranyldiphosphate biosynthesis II (via MEP) | 0.00412 | 0.40657 |
| PWY-6628 | C18:1/C18:2 | | ↑ | superpathway of phenylalanine biosynthesis | 0.00559 | 0.40657 |
| PWY-6629 | C18:1/C18:2 | | ↑ | superpathway of tryptophan biosynthesis | 0.00244 | 0.38197 |
| CYSTSYN-PWY | C18:1/C18:2 | | ↓ | cysteine biosynthesis I | 0.00583 | 0.28020 |
| PWY0-162 | C18:1/C18:2 | | ↓ | pyrimidine ribonucleotides *de novo* biosynthesis | 0.00627 | 0.28020 |
| PWY-3101 | C18:1/C18:2 | | ↓ | flavonol biosynthesis | 0.00216 | 0.22519 |
| PWY-3561 | C18:1/C18:2 | | ↓ | choline biosynthesis III | 0.00162 | 0.22519 |
| PWY-5060 | C18:1/C18:2 | | ↓ | luteolin biosynthesis | 0.00727 | 0.28453 |
| PWY-5129 | C18:1/C18:2 | | ↓ | sphingolipid biosynthesis (plants) | 0.00623 | 0.28020 |
| PWY-6605 | C18:1/C18:2 | | ↓ | adenine and adenosine salvage II | 0.00451 | 0.28020 |
| PWY-801 | C18:1/C18:2 | | ↓ | homocysteine and cysteine interconversion | 0.00140 | 0.22519 |
| SER-GLYSYN-PWY | C18:1/C18:2 | | ↓ | superpathway of serine and glycine biosynthesis I | 0.00855 | 0.29729 |
| GLYCOLYSIS | C18:2/C18:3 | | ↑ | glycolysis I | 0.00642 | 0.42142 |
| PWY0-163 | C18:2/C18:3 | | ↑ | salvage pathways of pyrimidine ribonucleotides | 0.00876 | 0.42142 |
| PWY-1042 | C18:2/C18:3 | | ↑ | glycolysis IV (plant cytosol) | 0.00786 | 0.42142 |
| PWY-5687 | C18:2/C18:3 | | ↑ | pyrimidine ribonucleotides interconversion | 0.00738 | 0.42142 |
| PWY-6619 | C18:2/C18:3 | | ↑ | adenine and adenosine salvage VI | 0.00542 | 0.42142 |
| BSUBPOLYAMSYN-PWY | C18:2/C18:3 | | ↓ | spermidine biosynthesis I | 0.00757 | 0.79023 |
| FOLSYN-PWY-1 | C18:2/C18:3 | | ↓ | superpathway of tetrahydrofolate biosynthesis | 0.00030 | 0.09377 |
| TRNA-CHARGING-PWY | C18:2/C18:3 | | ↓ | tRNA charging pathway | 0.00683 | 0.79023 |

^a^The FA ratio trait with the most significant *P* value. ^b^Additional FA ratio trait with significant *P* value (0.01 < *P* < 0.05). ^c^The pathway effect direction on FA ratio trait.↑, increasing FA ratio trait; ↓,decreasing FA ratio trait.
