# Supplementary material for: Leveraging GWAS data to identify metabolic pathways and networks involved in maize lipid biosynthesis
Source: Plant J. 2019 Mar 20;98(5):853–63. doi: 10.1111/tpj.14282 (PMC6850169; doi:10.1111/tpj.14282)
Supplement: Supplementary file 2 [file TPJ-98-853-s002.docx]

**SUPPORTING INFORMATION - LEGENDS**

**Table S1.** Summary of significantly associated genes from the GWAS study of Li et al., (2013), and those that were also identified by pathway analysis. Pathway identifier (ID) and name are drawn from the MaizeCyc database (https://www.maizegdb.org/metabolic_pathways/).

**Table S2.** Summary of significantly associated SNPs from the GWAS study of Li et al., (2013) with *R^2^* values, and the number of tagSNPs and genes from the present study.

**Table S3.** Pathways associated with increased FA content and with enrichment scores that were significant at 0.01 < *P* < 0.05. Pathway identifier (ID) and name are drawn from the MaizeCyc database (<https://www.maizegdb.org/metabolic_pathways/>), and *P* and FDR were calculated in this study.

**Table S4.** Number of genes which contributed to the enrichment score in significant pathways at *P* < 0.01, and which are shown as hatch marks at the top of the running enrichment score graphs. Pathway identifier (ID) and name are drawn from the MaizeCyc database (https://www.maizegdb.org/metabolic_pathways/).

**Table S5.** Pathways associated with decreased oil and FA concentration with enrichment scores significant at *P* < 0.05. Pathway identifier (ID) and name are drawn from the MaizeCyc database (<https://www.maizegdb.org/metabolic_pathways/>), and *P* and FDR were calculated in this study.

**Table S6.** Pathways associated with effects on the ratio of different FA traits with enrichment scores significant at *P* < 0.01. Pathway identifier (ID) and name are drawn from the MaizeCyc database (<https://www.maizegdb.org/metabolic_pathways/>), and *P* and FDR were calculated in this study. (<https://www.maizegdb.org/metabolic_pathways/>).
